# Molecular-scale in-operando reconfigurable electronic hardware†

**DOI:** 10.1039/d4nh00211c

**Published:** 2024-11-27

**Authors:** Yulong Wang, Qian Zhang, Cameron Nickle, Ziyu Zhang, Andrea Leoncini, Dong-Chen Qi, Alessandro Borrini, Yingmei Han, Enrique del Barco, Damien Thompson, Christian A. Nijhuis

**Affiliations:** a Department of Chemistry, National University of Singapore 3 Science Drive 3 117543 Singapore; b School of Chemistry and Chemical Engineering, Chongqing University Chongqing 400044 China; c Department of Physics, University of Central Florida Orlando Florida 32816 USA; d Centre for Materials Science, School of Chemistry and Physics, Queensland University of Technology Brisbane Queensland 4001 Australia; e Hybrid Materials for Opto-Electronics Group, Department of Molecules and Materials, MESA+ Institute for Nanotechnology, Molecules Center and Center for Brain-Inspired Nano Systems, Faculty of Science and Technology, University of Twente 7500 AE Enschede The Netherlands c.a.nijhuis@utwente.nl; f Department of Physics, Bernal Institute, University of Limerick Limerick V94 T9PX Ireland

## Abstract

It is challenging to reconfigure devices at molecular length scales. Here we report molecular junctions based on molecular switches that toggle stably and reliably between multiple operations to reconfigure electronic devices at molecular length scales. Rather than static on/off switches that always revert to the same state, our voltage-driven molecular device dynamically switches between high and low conduction states during six consecutive proton-coupled electron transfer steps. By changing the applied voltage, different states are accessed resulting in *in operando* reconfigurable electronic functionalities of variable resistor, diode, memory, and NDR (negative differential conductance). The switching behavior is voltage driven but also has time-dependent features making it possible to access different memory states. This multi-functional switch represents molecular scale hardware operable in solid-state devices (in the form of electrode–monolayer–electrode junctions) that are interesting for areas of research where it is important to have access to time-dependent changes such as brain-inspired (or neuromorphic) electronics.

## Abstract 2

A dynamic molecular switch with 3 different functionalities makes it possible to reconfigure devices at molecular length scales and to access different memory states by simply changing the applied voltage.

## Graphical abstract

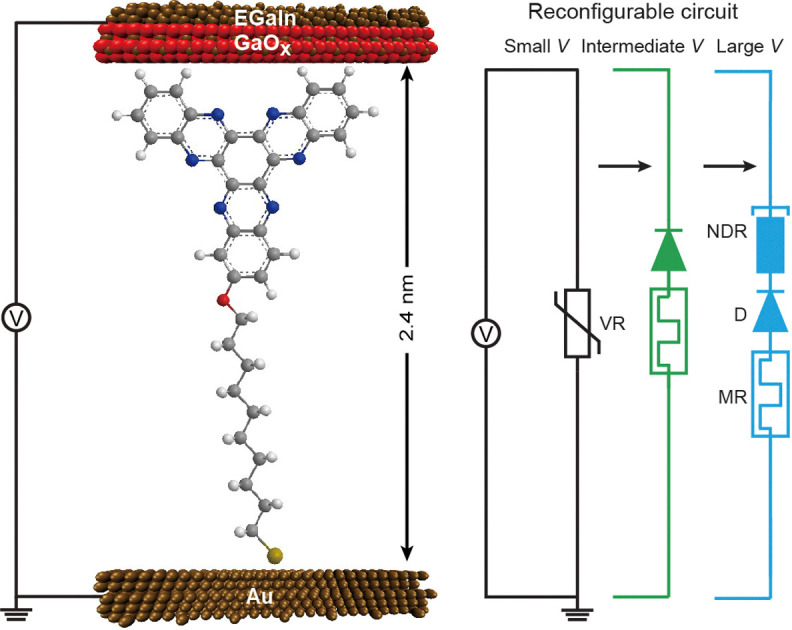

**New concepts**

Reconfigurable electronic hardware is promising to yield energy efficient circuits that are highly desirable for various applications including artificial intelligence or deep learning. Reconfigurable solid-state devices are known for decades, but reconfigurable molecular materials are needed for applications that require soft-materials, such as, wearable and stretchable electronics, soft robotics or healthcare applications. Molecular-scale devices, however, usually lack the stability because of spontaneous back switching (due to stochastic or thermal effects). We use the concept of proton coupled electron transport (PC-ET) enabled by reversible covalent N–H bond formation leading to stable switching. Here, the molecules change their chemical and associated electronic structure depending on the applied voltage. These changes in the molecular structure result in changes in electronic function including diode (with rectification ratios of 3.1 × 10^3^), memory (with on/off ratios of 2.5 × 10^3^), and negative differential resistance (with peak to valley ratios of 14.8). In contrast to other approaches, we demonstrate stable, molecular scale switching in devices consisting of a one-molecule thin layer of 2.4 nm. Our findings demonstrate that the concept of PC-ET can lead to reconfigurable molecular-scale devices and hopefully will inspire further development of other dynamical molecular systems to realize adaptive devices or energy efficient neuromorphic devices.

## Introduction

Devices based on traditional semiconductors are reaching their physical size limits and facing large power consumption.^1,2^ Devices with dynamic, run-time reconfigurability provides a potential path to overcome these challenges by allowing them to perform multiple tasks and reduce the need for additional components.^3^ Reconfigurable devices illustrate significant advantages in area efficiency and energy consumption,^4,5^ and they also simplify circuit design and make it more flexible, important features for many electronic applications, including neuromorphic computing^6,7^ and logic circuits.^8,9^ Unlike traditional devices with fixed hardware, reconfigurable devices are based on systems that change their physical properties by, for instance, stretching or deforming, or that can be dismantled and reassembled into different configurations.^10–12^ Such approaches are not practical for applications that require *in operando* (or electric field driven) reconfigurable systems that are very small (nano-scale). To further advance the rapidly evolving field of neuromorphic electronics, it is desirable to have access to devices with a temporal component, or time-dependent change in device configuration, and to demonstrate such operations at molecular length scales. Here, we report a molecular switch inside tunnel junctions that remembers its history of applied voltage and current. This results in hysteretic negative differential resistance (NDR) with a memory (*i.e.*, negative memristance) making it possible to reconfigure the junctions between a variable resistor, diode, and memory, *in operando*. Unlike previous static molecular switches that always switch between fixed values of the on and off states,^13–15^ the switching probability and the values of the on/off states continuously change depending on the applied bias voltage, demonstrating reconfigurable non-linear operations at the molecular length scale.

Reconfigurable solid-state devices based have been reported (based on ferroelectric^16,17^ or phase change materials^18,19^ for instance), but soft materials open the door to complementary applications including wearable, flexible and stretchable electronics, or healthcare applications.^20–23^ Soft molecular materials can be reconfigured due to changes in pressure (or deformation), temperature, relative humidity or pH, and they have been used for various applications, such as sensing, soft robotics, or bio-integrated electronics. Such systems, however, are large and usually based on elastomers combined with several other components, *e.g.*, liquid crystals, nanowires, or 2D materials.^3,11,24–26^ For example, Pan *et al.* reported electrically driven reconfigurable field effect transistors (FETs) based on van der Waals heterostructures (vdWHs) where the channel doping configuration and carrier injection into the channel could be controlled.^6^ In general, however, devices cannot be reconfigured *in operando*. For instance, Tao *et al.* demonstration of reconfigurable FETs required assembly and disassembly steps to alter the layer sequencing of the vdWHs,^26^ and Qiu *et al.* demonstrated a reconfigurable molecular device where the magnitude and direction of current rectification could be controlled by exposing the junctions to different solutions of monolayer precursors.^10^ One of the reasons why it is challenging to obtain reconfigurable systems at the molecular length scale is the stochastic switching frequently observed in single molecule junctions,^27,28^ and also present in large-area junctions^29–33^ and in thin film devices.^34^ Here, the on or off states are unstable and spontaneously switch back, resulting in loss of memory state, irregular turn on and off voltages, or abrupt changes in current, and leading to asymmetrical (or even triangular) NDR peaks.^28,35^ Such instabilities may indicate switching due to artefacts (*e.g.*, filaments or water).^36^

To go beyond the current strategies and create reconfigurable electronic devices operable at the molecular length scale, we developed a voltage-driven molecular switch inside tunnel junctions. Our *in-operando* reconfigurable system does not require geometrical or structural changes of the device, exposure to solutions, or dismantling/re-assembling steps. The operating mechanism is fully modelled and involves proton coupled electron transport (PC-ET). The different configurations of our junctions are stabilized by dynamic covalent N–H bond formation, which can effectively eliminate stochastic behavior and result in symmetrical, stable NDR peaks with large hysteresis that provides memory function. Unlike previous static molecular switches that always switch between fixed values of the on and off states, or our earlier findings on time-dependent switching to emulate synaptic behavior,^37^ this reconfigurable system represents a new class of switch. The on/off states continuously change depending on the applied bias and making it possible to access different electronic functionalities (*e.g.*, 1 diode–1 resistor random access memory, 1D–1R RAM, a very desirable feature to reduce sneak path currents in crossbar arrays^29,38^). All the characteristics are achieved within a 2.4 nm, or one molecule thick, layer with low operating voltage of 2 V. Finally, we demonstrate that the time-dependent switching characteristics allow us to access three different memory states at three different read-out times.

## Results and discussion

### Operation principle of molecular-scale reconfigurability

**Fig. 1:**
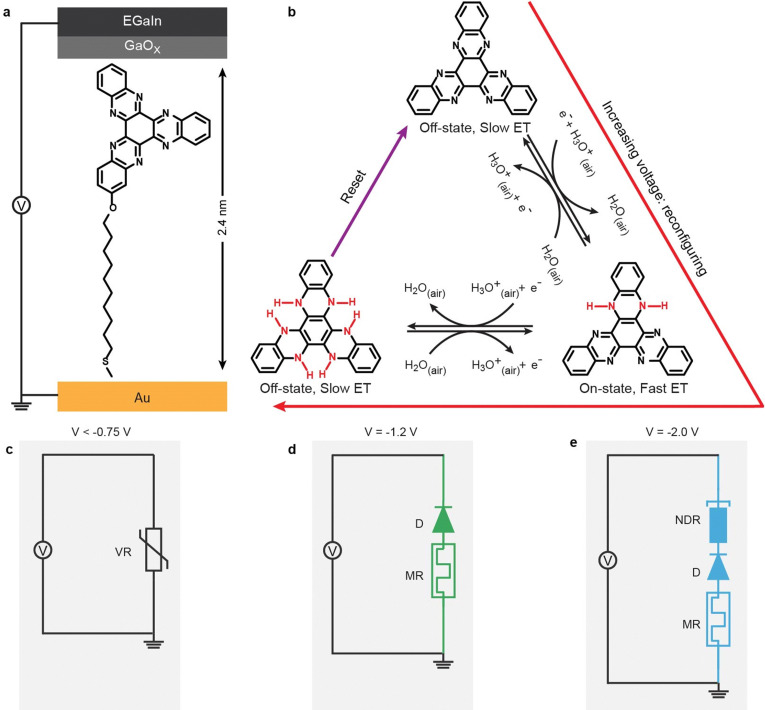
Schematic illustrations of the reconfigurable junction. (a) the structure of HATNA molecular junction; (b) the reconfigurable process of the HATNA moiety *via* proton coupled electron transfer (PC-ET) mechanism: in the initial state the HATNA moiety is fully oxidized form *via* a slow electron transfer (ET) for coherent tunneling, at intermediate voltages the HATNA moiety is the partially reduced state *via* a fast ET for coherent tunneling, at large voltages the HATNA moiety is the fully reduced state *via* a slow ET again for coherent tunneling. The red and violate arrows indicate the reconfigurable and reset processes of the HATNA junction. The corresponding equivalent circuits indicate that the junction can be reconfigured to a variable resistor (VR, panel c), 1D–1R memory element (D = diode and MR = memristor, panel d), and negative differential resistance (NDR) in series with memristor and diode (panel e). The top electrode is fabricated *via* the EGaIn technique^40^ with a self-limiting thin oxide layer^41^ of 2–3 nm (GaO_*x*_) covering the surface of the EGaIn alloy (eutectic alloy of Ga and In), and the bottom electrode is fabricated with template-stripped Au. The thickness of the HATNA SAM is 23.6 ± 1.0 Å (see Materials and Methods), as indicated with the black solid arrow in panel a.

Fig. 1 shows a schematic illustration of the reconfigurable junction consisting of self-assembled monolayers (SAMs) with 5,6,11,12,17,18-hexaazatrinaphthylene (HATNA) terminal groups in contact with Au and eutectic gallium–indium (EGaIn) electrodes. These HATNA moieties readily undergo six proton coupled electron transfer (PC-ET) steps^39^ and the switching mechanism of the molecules in junctions has been studied by us in detail and reported in ref. 37 Depending on the oxidation state of H_*n*_-HATNA (where n indicates the extent of reduction), the electron transfer (ET) rate by coherent tunneling across the junction is fast (fast ET) in the on state, or slow (slow ET) in the off state. The N–H bond formation stabilizes the oxidation state of HATNA “locking” the junction in a state with fast or slow ET and associated functionalities of variable resistor (VR) at low voltages with HATNA in the fully oxidized form (Fig. 1b and c), then diode (D) and memory (MR) functionality (1D–1R RAM) at intermediate voltages with HATNA in partially reduced state (Fig. 1b and d), and followed by negative differential resistance (NDR) functionality at large voltages with HATNA in highly reduced state (Fig. 1b and e). The entire system can be reset to the original state by oxidation of HATNA at opposite bias. Thus, this dynamic N–H bond formation locks the different oxidation states and associated electronic configuration, which sets the energy level alignment of the junction and so defines electronic function (as explained in detail below). In other words, the junctions are reconfigured at the atomistic length scale by reversible N–H bonds to create stable configurations simply by changing the applied voltage bias. This approach of changing molecular states *via* applied voltage gives reconfigurable junctions, with different functionalities from variable resistor to 1D–1R RAM to hysteretic NDR. In our earlier work we showed that the time (as distinct from voltage) dependent switching response of these junctions exhibited spike-timing dependent switching akin to synapses. These junctions are extremely stable and can be switched for 1 × 10^5^ times.^37^

### Electrical characterization of the junctions

**Fig. 2:**
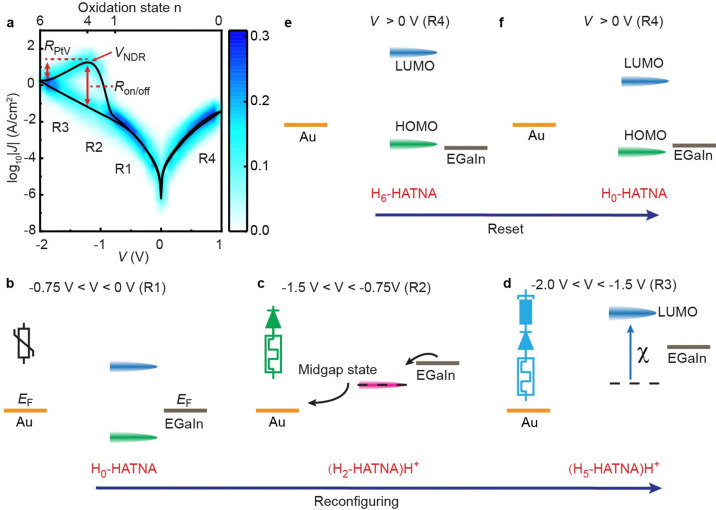
Operating mechanism of reconfigurability. (a) Heatmap of the log_10_|*J*| *vs. V* consisting of 171 *J*(*V*) curves recorded from 57 junctions in ambient environment (24 °C, relative humidity = 60%) obtained with a yield of non-shorting junctions of 90.5%. The black line is log-median average curve (<log_10_|*J*|>_m_*vs. V*). The peak-to-valley ratio (*R*_PtV_) is defined as the peak current at peak voltage (*V*_NDR_) divided by the valley current, and the current on/off ratio (*R*_on/off_) is the current at forward bias divided by current at reverse bias at *V*_NDR_. Regions R1–4 indicate the voltage regions corresponding to the energy level diagrams in panels b–f along with corresponding HATNA structures (insets) with (b) HATNA in the fully oxidized form, (c) partially reduced HATNA and (d) almost fully reduced HATNA. At positive voltages, HATNA oxidizes back to its original state and reconfiguring can be repeated (e) and (f).

**Fig. 3:**
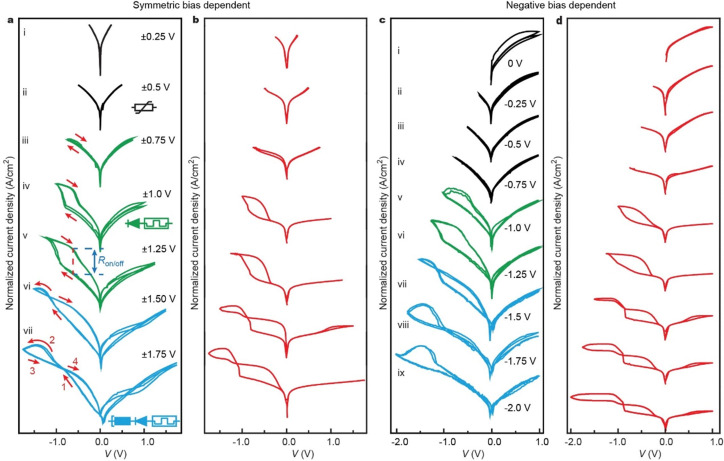
Reconfigurable behavior of the junctions. (a) Bias window dependent measurement of the junction with symmetric scans ranging from ±0.25 V to ±1.75 V. The insets show the equivalent circuits of a variable resistor (curve ii), variable resistor in series with diode (1D–1R memory, curve iv), and NDR in series with 1D–1R (curve vii). (b) Calculated *I*–*V* cycles (red) for the corresponding symmetric bias cycles. (c) Negative voltage dependent measurement keeping maximum positive bias at +1.0 V. (d) Calculated *I*–*V* cycles (red) for the corresponding negative bias dependent cycles. See Fig. S6 to S10 (ESI†) for additional data sets.

**Fig. 4:**
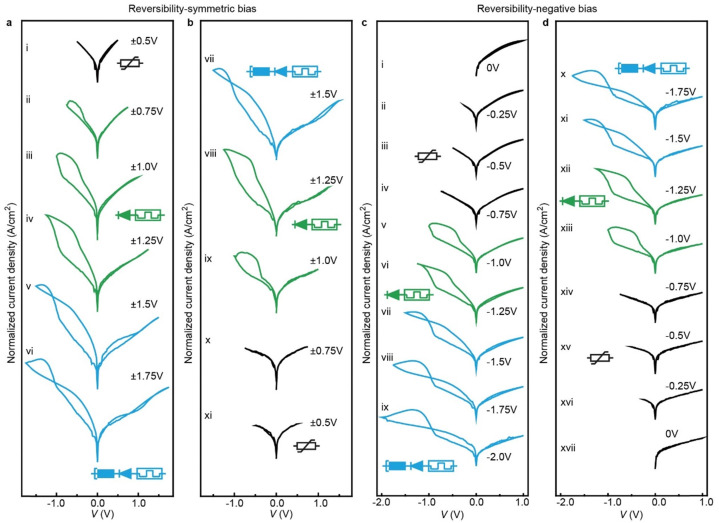
Demonstration of reversible reconfiguration of the junctions. (a) and (b) with symmetric voltage dependent measurements: (a) the bias windows increased from ±0.5 V to ±1.75 V, and (b) then back to ±0.5 V. (c) and (d) with negative voltage dependent measurements: (c) keeping the positive voltage at 1.0 V and the negative voltage changing from 0 V to −2.0 V, and (d) then back to 0 V. Keeping the maximum voltage similar to the junction shown in Fig. 3, with increasing applied bias window the junction is reconfigured and the entire process is reversible with decreasing applied bias window and the junction can be reconfigured back to its original state (additional datasets of the reversible reconfigurability are shown in Fig. S11, ESI†). These experiments were performed continuously under consistent conditions of room temperature (25 °C) and humidity (RH = 60%).

We derived the HATNA functionalized SAMs on Au from the thioacetate derivative and formed junctions by contacting the monolayers with EGaIn (eutectic Ga-In alloy) as described recently (see ESI† Section S6 for details).^37^Fig. 2a shows the heatmap of the *J*(*V*) response of Au–S–C_10_-HATNA//GaO_*x*_/EGaIn junctions in the applied bias window of *V* = −2.0 V to 1.0 V. This heatmap clearly shows the three electronic features of our junctions: diode, memory, and NDR. The individual *J*(*V*) traces are smooth and the NDR peak is symmetrical (see Fig. 3 and 4 below, Fig. S5–S9, and Fig. S11, S13, ESI†). These features indicate that our switches avoid stochastic transitions characterized by excessive noise, or triangular peak shapes, as frequently observed in single molecule junctions as well as in large-area junctions,^27–29,35,42,43^ or memristors based on filaments,^44^ or junctions that suffer from artefacts.^36^ Unlike resonant tunneling diodes,^45^ our junctions show pronounced hysteresis which is a desirable feature for memory applications^46,47^ or neuromorphic computing.^37^Fig. 2a also defines the NDR peak position (*V*_NDR_), peak-to-valley ratio (*R*_PtV_), and current on/off ratio (*R*_on/off_, current at forward bias divided by current at reverse bias at *V = V*_NDR_). The value of *R*_PtV_ = 14.8 ± 2.8 is comparable to state-of-the-art molecular resonant tunnel diodes^37,48^ and two times larger than the value reported by the group of van der Zant for a molecular junction with stable NDR.^48^ The value of *V*_NDR_ is −1.22 V, a desirable feature as the operating voltage remains within 2 V. The value of *R*_on/off_ is (2.80 ± 1.15) × 10^2^ for our junctions. This value is the highest reported for hysteretic NDR devices and is large for molecular memory devices in general.^29,37^ Finally, the diode functionality is characterized by dividing the forward current by the reverse current at a given voltage. This diode functionality is a direct consequence of the asymmetry of the S–C_10_-HATNA molecule where the HATNA functionality is in direct contact with the top electrode but separated from the bottom electrode by the long alkyl chain resulting in asymmetrical potential drops along the molecule similar to other types of redox-active junctions.^49,50^ The diode properties are discussed below in more detail.

Fig. 2 also shows the mechanism of reconfigurability of the junctions. At low voltages (Fig. 2b), the HATNA moieties are in their fully oxidized form (H_0_-HATNA) and have a large HOMO–LUMO gap (HOMO = highest occupied molecular orbital, LUMO = lowest unoccupied molecular orbital). In this configuration, the LUMO and HOMO are far away from the Fermi-levels (represented by *E*_F_) of the electrode and, therefore, they cannot enter the conduction window. Thus, the junctions behave as variable resistors where the mechanism of charge transport is dominated by off-resonant tunneling (indicated by region 1 (R1) in Fig. 2a). At intermediate voltages, PC-ET occurs reconfiguring the junctions to H_*n*_-HATNA with *n* = 1–4 (Fig. 2c). In this configuration, a new inter gap state forms through which charges can effectively tunnel turning the devices on.^37^ This leads to an increase in *J* indicated as region 2 (R2) in Fig. 2a. Increasing the voltage further reconfigures the molecules again to H_*n*_-HATNA with *n* = 5–6 (Fig. 2d) which leads to a decrease in *J* as indicated as region 3 (R3) in Fig. 2a. In this configuration, the inter gap state disappears again turning the devices off and the mechanism of charge transport reverts to off-resonant tunneling. This switching between HATNA configurations leads to NDR. The HATNA configuration can be reset at opposite voltage (R4) when the HOMO of the HATNA moieties enter the bias window (Fig. 2e) and the molecules can be oxidized back to their initial configuration (Fig. 2f). This leads to the hysteric NDR seen in Fig. 2a that forms the basis for memory. The NDR peak is very well resolved at negative applied bias in Fig. 2a, but not at positive bias. For large applied voltages of 1.5 V or 1.75 V, the hysteresis and NDR peak become also visible at positive bias (see for example Fig. 3a curves vi or vii, or Fig. S10, ESI†).

### Reconfigurable operation of the devices

To demonstrate that the HATNA junctions can be reconfigured to specific electronic functions *in operando* (as depicted in Fig. 1), we changed the applied bias window to control the oxidation state of HATNA as shown in Fig. 3a with additional data sets are given in Figs. S6 to S10 (ESI†). Fig. 3a shows that for small bias windows of <±1.0 V, typical off-resonant tunneling behavior is observed (see ESI† Section S6.4) akin to a variable resistor (inset to sub-panel ii). In this bias regime, no molecular levels can enter the bias window Fig. 2b. At moderate bias windows of ±1.0 to ±1.5 V (Fig. 2c), the HATNA is only partially reduced (*n* = 1,2) which locks the junction in the on state resulting in a memory switch with *R*_on/off_ = 8.1 × 10^2^ at *V* = −0.63 V (Fig. 3a curve v). Here, the junction was reconfigured from variable resistor to memory element by controlling applied bias rather than changing the molecular structure *ex situ*, so we avoided dismantling and reassembly of the junctions.^29,51,52^ In this configuration, the current is effectively blocked at positive voltages resulting in a distinct 1D–1R RAM characteristic demonstrating that the molecular asymmetry results in effective diode behavior. The current rectification ratio RR = |*J*(−1.2 V)/*J*(1.2 V)| = 3.1 × 10^3^. This combination of large *R*_on/off_ and RR with such a low operating voltage is to the best of our knowledge, state of the art.^29^ At large bias windows of > ±1.5 V (Fig. 2d), the junction is reconfigured again and the NDR region is accessible (Fig. 3a curve vii). At this large bias, the HATNA approaches full reduction and therefore the junction switches off again.

To demonstrate that the junctions can be reset only at positive bias when the HOMO enters the bias window (Fig. 2e–f), we subjected the junctions to 0 V→−2 V→0 V sweeps. Only the first voltage cycle showed pronounced NDR feature after which the junction switched off and remained off in subsequent voltage cycles (Fig. S13a curve i, ESI†) because the HATNA remains fully reduced (Fig. 2e). With increasing positive bias (Fig. S13a curves ii–vii, ESI†), the junction progressively turns back on again restoring the NDR feature because the HATNA moieties are oxidized back to their initial state. However, the threshold voltage to trigger the reset process in the junctions is not specific. With a small positive voltage (*e.g.*, +0.25 V), the junctions can be partially reset and only a minor NDR observed. As the positive voltage increases, the junctions can completely reset in the range of +0.50 V to +0.75 V and the maximum NDR is observed.

By limiting the negative bias window to −1.2 V and controlling the applied positive bias, the junctions can be turned on in varying degrees because the NDR region is not accessible. Fig. S13c (ESI†) shows that for 0 V → −1.2 V → 0 V sweeps the junctions do not turn on, but with increasingly larger positive bias the junction resets and progressively turns on at negative bias (due to partial reduction of H_*n*_-HATNA with *n* = 1 or 2) reaching a maximum value of *R*_on/off_ = 2.5 × 10^3^ at *V* = −0.56 V (Fig. S13c curve viii, ESI†). In this state, junctions have large current rectification ratio of RR = |*J*(−1.2 V)/*J*(1.2 V)| = 3.1 × 10^3^ thus exhibiting both diode (*D*) and variable resistor (*R*) functionalities in series reminiscent of 1D–1R RAM. We would like to emphasize that these 1D–1R RAM characteristics belong to the best performing junctions reported to date despite the low drive voltages.^29,53–55^ Similarly, an optimal hysteretic NDR region can be found by keeping the maximum positive voltage constant (*e.g.*, +1.0 V as in Fig. 3c) and varying the negative bias window. This reconfigurable behavior is captured by the theoretical model (Section S7, ESI†) as discussed in the next section. Interestingly, the junctions show distinct crossing, or crossed loops, in, for example, Fig. 3a for curves vi and vii. In these conditions, the voltage at negative bias is not large enough to fully turn the molecular switches off and they remain partially in the on-state during the backward trace. Therefore, the currents during the backward trace (below the NDR peak) remain higher than during the forward trace. At positive bias, the molecules are oxidized back to the off-state meaning that during the return the currents are lower resulting in crossed loops.

### Modelling of reconfigurable behavior

We modelled the reconfigurable behavior of the junctions using a theoretical model developed by Migliore and Nitzan^42^ which was further parametrized by molecular density functional theory (DFT) as described in our previous report.^37^ Here, the tunneling currents across the junction in the on and off states are described by Landauer-Büttiker theory and the transitions due to proton addition steps are modelled by Marcus theory. Fig. 3b, d and Fig. S13b, d (ESI†) show the corresponding modelled *I*(*V*) curves (red; fitting details are given in Section S7, ESI†). While keeping the majority of the parameters constant across the 32 different data sets, the model accurately describes the four main characteristics of the junctions. First, a typical reversible off-resonant tunneling behavior akin to a variable resistor for voltages cycles below ±1.0 V with HATNA in the configuration shown in Fig. 2b. Second, a molecular memory switch with a ∼10^3^ on/off ratio for moderate voltage cycles between ±1.0 and ±1.25 V with HATNA in the configuration shown in Fig. 2c. The third feature is the hysteretic NDR for voltage cycles above ±1.25 V with HATNA in the configuration shown in Fig. 2d. Finally, the model also accounts for the diode functionality for voltages cycles above ±1.0 V. Our model confirms that the molecular reduction occurs at negative bias, while at positive bias the molecule is oxidized back to its original molecular state. The model fully retrieved the experimental data in Fig. S13b (ESI†) and confirmed that at positive bias the junction can only be reset (*i.e.*, configuration from H_6_-HATNA to H_0_-HATNA) when the HOMO falls in the conduction window.

### Demonstration of reversible reconfigurability

To demonstrate that all transitions are reversible, we increased the bias window followed by a decrease of the bias window of the same junction. Fig. 4a and b shows that, as in Fig. S11 (ESI†), the junction at low applied bias behaves as a variable resistor (curve i), then the junction is reconfigured to a 1D–1R RAM element at intermediate applied bias (curves ii–iv), and finally the junction reconfigures to the NDR regime at large applied bias (curves v and vi). As the applied bias is reversed, the junctions sequentially display an NDR effect, 1D–1R memory and variable resistor again (curves vii to xi). The applied voltage can be varied to optimize electrical function of the junction as needed. For instance, Fig. 4c and d shows a voltage sequence where only the negative applied voltage was changed while keeping the positive applied voltage at *V* = 1.0 V to maximize the NDR feature (sequence i to ix). Also in this case, the entire process is reversible, and the junction reconfigures back to its original state by reducing the applied bias (sequence x to xvii).

### Switching of dynamic memory

**Fig. 5:**
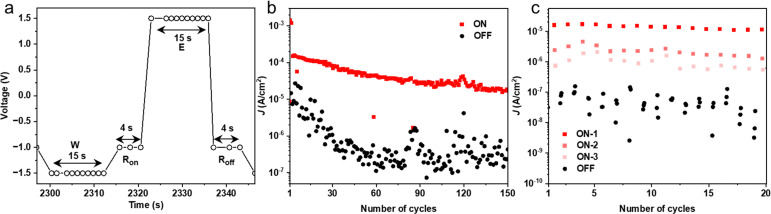
Switching of the junctions. (a) the WRER pulse sequence, (b) the endurance over 150 WRER cycles, (c) the output current of the dynamic junction.

Although this work focuses on the voltage driven changes in the junctions, previously we have shown that the HATNA molecules also switch in a time-dependent manner akin to synapses.^29^ We show here that this time-dependent behavior can be used to access different memory states as follows. Fig. 5 shows write–read–erase–read (WRER) voltage sequence and current response with the junctions in the on state after *W* (*R*_on_ in red) or off state after *E* (*R*_off_ in black). The value of *R*_on_ was measured at three different time-intervals over a period of time of 4 s (See Fig. S14 for additional data, ESI†) for 150 cycles (Fig. 5b). Fig. 5c shows an excerpt of 20 cycles along with the three states marked by the three horizontal dashed lines. The junctions switch between the on-state and off-state with high on/off ratios of two orders of magnitude for WRER measurements (ON-1 in Fig. 5c), and the two other read-out states at 2 s and 4 s delay (ON-2 and ON-3 in Fig. 5c) are well-resolved but with smaller on/off ratios demonstrating the time-dependent nature of this type of memory. It is important to note that depending on the read-out time a new, but smaller, memory state can be accessed; this behavior is repeated during the next cycle demonstrating that the junction can be fully restored, and all changes are reversible. Similarly, Fig. 5b shows that the on/off ratio increases during the first WRER cycles, but after about 40 cycles the junction reaches a steady-state where the on/off ratios are constant for a given time delay during consecutive cycles. Such time dependent features with the ability to access different memory states are desirable for applications where time-dependence is important, *e.g.*, reservoir computing.^56,57^

## Conclusions

In summary, here we report a new type of molecular-scale reconfigurable two-terminal device of only 2.4 nm thickness. This molecular device can be reconfigured *in operando* between variable resistor, diode, and different types of memory (hysteretic NDR with *R*_on/off_ = 2.80 × 10^2^ and *R*_PtV_ = 14.8, and 1D–1R memory with RR = 3.1 × 10^3^ and *R*_on/off_ = 2.5 × 10^3^; amongst the highest values reported for molecular-scale devices). The mechanism that drives the reconfiguration of the devices has been fully modelled showing the dynamic covalent bond formation resulting in stable, multi-functional behavior that stands in stark contrast to typical (hysteretic) on/off switches reported so far. Although this work focusses on the voltage driven changes of the junctions, the junction characteristics also have a time-dependent feature making it possible to access different memory states at different read out times. In principle, this time-dependent behavior should not only be dependent on the read out times, but also on the chemical environment (including relative humidity or pH) of the molecules since the mechanism of charge transport is governed by proton-coupled electron transfer reactions which we are currently investigating.^58^ We hope that our demonstration of reconfigurable molecular junctions will inspire new lines of research to develop other types of dynamical molecular systems, *e.g.*, to realize adaptive neuromorphic devices.^37,59–61^

## Materials and methods

### Synthesis

The SAMs were derived from a 5,6,11,12,17,18-hexaazatrinaphthylene (HATNA) thioacetate derivative that was synthesized and characterized as reported in our recent work.^37^ Section S1 (ESI†) gives a brief summary.

### Self-assembled monolayer (SAM) formation

The HATNA SAMs were prepared following a previous method.^62^ We dissolved 1.0 mg AcS-C_10_-HATNA in 5 ml freshly distilled tetrahydrofuran (THF, with a concentration of 0.33 M), then the solution was flushed with N_2_ for about 15 min to remove the oxygen. After that, 20 μl ammonia (26–28%) was added, and freshly prepared Au surfaces were immersed in the solution. After about 24 h, the Au substrates were taken out from the solution and washed immediately with about 30 ml THF and ethanol, and then the substrates were gently dried with N_2_ flow.

### Electrode preparation

The top electrode was prepared with the EGaIn technique. Here, an EGaIn alloy to form a cone shape tip is used which is then used to form contacts with the SAMs as described in detail in previous reports.^63^ The bottom electrode was fabricated using the template-stripping generating surfaces that are clean and flat with typical root mean square surfaces roughness of 2–3 Å over an area of 1 × 1 μm^2^.^64,65^ First, a 200 nm thick Au (99.999% Au granules, *ACI* Alloys) thin film was deposited on a Si wafer (with a native SiO_2_ thin layer on surface, Syst Integration Pte Ltd) using a thermal evaporator (Shen Yang Ke Yi), then pre-cleaned glass slides were adhesive on the Au surface with thermal glue (EPOTEK 353ND). After that, the Au thin film was heated at 80 °C for 3 hours to cure the adhesive. The Au electrode was stored in a dry box and template-stripped immediately before use.

### Electrochemistry

The cyclic voltammetry measurements of the HATNA SAMs were conducted with an Autolab PGSTAT302T setup equipped with NOVA 1.10 software. In the measurements, a Pt plate was worked as the counter electrode, an Ag/AgCl electrode was used as the reference electrode, the Au substrate coating with SAM was supported as the work electrode, and A HClO_4_ aqueous solution (2M) was used as the electrolyte. We recorded the results between −0.4 V and 1.0 V with various scan rates from 0.02 V s^−1^ to 100 V s^−1^ (Section S4, ESI†).

### Surface characterization

The surface characterizations of the HATNA SAMs were performed with X-ray photoelectron spectroscopy (XPS) and ultraviolet photoelectron spectroscopy (UPS), and near-edge X-ray absorption fine structure (NEXAFS) spectroscopy, which were supported by the Surface, Interface and Nanostructure Science (SINS) beamline of the Singapore Synchrotron Light Source (SSLS). The measurements were conducted on template-stripped Au surfaces coated with S–C_10_-HATNA SAM, with similar procedures in our previous work.^66^ In the high-resolution XPS measurements, the photon energy was optimized to obtain the optimal signals according to different elements, with the value at 350 eV for S 2p and C 1s, 600 eV for N 1s and 650 eV for O 1s spectra. All the XPS measurements were recorded at two different photoelectron take-off angles with values of 90° (normal emission, NE) and 40° (grazing emission, GE). By removing the background with the Shirley method, the XPS peaks were fitted with a fixed ratio of Gaussian (70%) and Lorentzian (30%) based on the *pseudo*-Voigt functions. The detailed results of surface characterization are shown in S5 (ESI†).

### Junction fabrication and electrical measurements in air

The junctions were fabricated with a grounding Au bottom electrode, a HATNA monolayer, and a cone-shaped GaO_*x*_/EGaIn top electrode, in which the bias was applied on the top EGaIn electrode, similar to the previous report.^63^ The geometrical footprint of the EGaIn top-electrode with the monolayers was 300–700 μm^2^. The electrical measurements were carried out with a LabVIEW-controlled Keithley 6430 source meter. We recorded the *J*(*V*) curves with voltage from 0 V→ +1 V → 0 V → −2 V → 0 V in steps of 20 mV (Section S6, ESI†).

### Bias window dependent experiments

We measured the junctions at different bias window at scan rate of 10 mV s^−1^, including symmetric voltage scan, positive voltage dependent measurement and negative voltage dependent measurement. For the symmetric bias window dependent measurement, we measure the *J*(*V*) curves at ±0.25 V, ±0.50 V, ±0.75 V, ±1.0 V, ±1.25 V, ±1.50 V and ±1.75 V. For the positive voltage dependent measurement, we control the negative at −2.0 V (or at −1.2 V), and change the positive voltage at 0 V, 0.25 V 0.50 V, 0.75 V, 1.0 V, 1.25 V, 1.50 V and 1.75 V. In the negative voltage dependent measurement, we set the positive voltage at 1.0 V, and adjust the negative voltage at 0 V, −0.25 V, −0.50 V, −0.75 V, −1.0 V, −1.25 V, −1.50 V, −1.75 V and −2.0 V (Section S6, ESI†). For the reversible reconfigurability experiment, we measure the *J*(*V*) curves from ±0.50 V, ±0.75 V, ±1.0 V, ±1.25 V, ±1.50 V to ±1.75 V, and then scan backward with the same voltage windows in reverse order. Or keep the positive voltage at +1.0 V, and increase the negative from 0 V, −0.25 V, −0.50 V, −0.75 V, −1.0 V, −1.25 V, −1.50 V, −1.75 V to −2.0 V and then scan backward with the same voltage windows in reverse order (as shown in Fig. 4 and Fig. S11, ESI†).

### Statistical analysis of *J*(*V*) characteristics

The statistical analysis of *J*(*V*) data was performed with the median averages of the current (<log_10_|*J*|>_m_) and the median absolute deviations (*σ*_m_), as recommended in the previous report,^67^ because this method does not reply on presumptions regarding the type of data distribution as explained in more detail in ref.37 The values of <log_10_|*J*|>_m_ and *σ*_m_ were calculated from the log_10_|*J*| values measured at each bias step. The heatmaps of the *J*(*V*) curves were created using OriginPro 2019b software with 2D kernel density estimations. In the 2D kernel density estimations, the density values were calculated based on a bi-dimensional Gaussian kernel, and the bandwidth was selected through a bivariate kernel density estimator with a grid size of 100.

### Theoretical modelling of the transport dynamics

We analyzed the switching behaviour of the junctions using a theoretical model developed by Migliore and Nitzan.^42^ The current going through the junctions for both the on and off conduction states were accounted with the standard Landauer single-level quantum model but adjusted to describe currents across large-area junctions.^68^ The fitting curves for the junctions have been presented in Fig. 3 and Fig. S13 (ESI†). The detailed description of the modelling has been provided in Section S7 (ESI†).

## Author contributions

Y. W., Q. Z. and C. N. contributed equally to this work. C. A. N. conceived and supervised the project. E. d. B. and C. N. conducted the numerical modelling. Z. Z., Y. H. and D.-C. Q. performed the surface characterizations. Y. W., Q. Z. and A. B. performed the electrical measurements. A. L. performed the Origin code data analysis. C. A. N., E. d. B., D. T. wrote the manuscript and all the authors commented on it.

## Data availability

Data are available in the supplementary materials and source data is available on https://dataverse.harvard.edu/privateurl.xhtml?token=9918ab97-db54-4cde-b334-6f8ac559b1bd.

## Conflicts of interest

The authors declare no competing financial interest.

## Supplementary Material

NH-010-D4NH00211C-s001

## References

[cit1] Lundstrom M. (2003). Science.

[cit2] Keyes R. W. (2005). Rep. Prog. Phys..

[cit3] Fei W., Trommer J., Lemme M. C., Mikolajick T., Heinzig A. (2022). InfoMat.

[cit4] Trommer J. , Heinzig A., Baldauf T., Mikolajick T., Weber W. M., Raitza M. and Völp M., Reconfigurable nanowire transistors with multiple independent gates for efficient and programmable combinational circuits, 2016 Design, Automation & Test in Europe Conference & Exhibition (DATE), 2016, pp. 169–174

[cit5] Rai S., Trommer J., Raitza M., Mikolajick T., Weber W. M., Kumar A. (2019). IEEE Trans. Very Large Scale Integr. VLSI Syst..

[cit6] Pan C., Wang C.-Y., Liang S.-J., Wang Y., Cao T., Wang P., Wang C., Wang S., Cheng B., Gao A., Liu E., Watanabe K., Taniguchi T., Miao F. (2020). Nat. Electron..

[cit7] Xu M., Chen X., Guo Y., Wang Y., Qiu D., Du X., Cui Y., Wang X., Xiong J. (2023). Adv. Mater..

[cit8] Roe D. G., Park S. H., Jeong S. Y., Choi Y. Y., Ahn J.-H., Woo H. Y., Cho J. H. (2024). Adv. Funct. Mater..

[cit9] Ram A., Maity K., Marchand C., Mahmoudi A., Kshirsagar A. R., Soliman M., Taniguchi T., Watanabe K., Doudin B., Ouerghi A., Reichardt S., O’Connor I., Dayen J.-F. (2023). ACS Nano.

[cit10] Qiu X., Rousseva S., Ye G., Hummelen J. C., Chiechi R. C. (2021). Adv. Mater..

[cit11] Zhu J., Dexheimer M., Cheng H. (2017). npj Flexible Electron..

[cit12] Kang J., Son D., Vardoulis O., Mun J., Matsuhisa N., Kim Y., Kim J., Tok J. B. H., Bao Z. (2019). Adv. Mater. Technol..

[cit13] Zhang J. L., Zhong J. Q., Lin J. D., Hu W. P., Wu K., Xu G. Q., Wee A. T. S., Chen W. (2015). Chem. Soc. Rev..

[cit14] Xin N., Guan J., Zhou C., Chen X., Gu C., Li Y., Ratner M. A., Nitzan A., Stoddart J. F., Guo X. (2019). Nat. Rev. Phys..

[cit15] Chen H., Stoddart J. F. (2021). Nat. Rev. Mater..

[cit16] Liu X., Ting J., He Y., Fiagbenu M. M. A., Zheng J., Wang D., Frost J., Musavigharavi P., Esteves G., Kisslinger K., Anantharaman S. B., Stach E. A., OlssonIII R. H., Jariwala D. (2022). Nano Lett..

[cit17] Liu Q., Cui S., Bian R., Pan E., Cao G., Li W., Liu F. (2024). ACS Nano.

[cit18] Zhang Y., Fowler C., Liang J., Azhar B., Shalaginov M. Y., Deckoff-Jones S., An S., Chou J. B., Roberts C. M., Liberman V., Kang M., Ríos C., Richardson K. A., Rivero-Baleine C., Gu T., Zhang H., Hu J. (2021). Nat. Nanotechnol..

[cit19] Chen X., Zhang S., Liu K., Li H., Xu Y., Chen J., Lu Y., Wang Q., Feng X., Wang K., Liu Z., Cao T., Tian Z. (2022). ACS Photonics.

[cit20] Lee G.-H., Moon H., Kim H., Lee G. H., Kwon W., Yoo S., Myung D., Yun S. H., Bao Z., Hahn S. K. (2020). Nat. Rev. Mater..

[cit21] Mackanic D. G., Chang T.-H., Huang Z., Cui Y., Bao Z. (2020). Chem. Soc. Rev..

[cit22] Zhang Z., Bao Z. (2023). Natl. Sci. Rev..

[cit23] Kim J., Yoo S., Liu C., Kwak S. S., Walter J. R., Xu S., Rogers J. A. (2023). Nat. Rev. Bioeng..

[cit24] Kaspar C., Ravoo B. J., van der Wiel W. G., Wegner S. V., Pernice W. H. P. (2021). Nature.

[cit25] Sun X., Zhu C., Yi J., Xiang L., Ma C., Liu H., Zheng B., Liu Y., You W., Zhang W., Liang D., Shuai Q., Zhu X., Duan H., Liao L., Liu Y., Li D., Pan A. (2022). Nat. Electron..

[cit26] Tao Q., Wu R., Li Q., Kong L., Chen Y., Jiang J., Lu Z., Li B., Li W., Li Z., Liu L., Duan X., Liao L., Liu Y. (2021). Nat. Commun..

[cit27] Jia C., Migliore A., Xin N., Huang S., Wang J., Yang Q., Wang S., Chen H., Wang D., Feng B., Liu Z., Zhang G., Qu D.-H., Tian H., Ratner M. A., Xu H. Q., Nitzan A., Guo X. (2016). Science.

[cit28] Schwarz F., Kastlunger G., Lissel F., Egler-Lucas C., Semenov S. N., Venkatesan K., Berke H., Stadler R., Lörtscher E. (2016). Nat. Nanotechnol..

[cit29] Guo Y., Wang B., Zhang X., Yuan S., Ma L., Wang J. (2020). InfoMat.

[cit30] Blum A. S., Kushmerick J. G., Long D. P., Patterson C. H., Yang J. C., Henderson J. C., Yao Y., Tour J. M., Shashidhar R., Ratna B. R. (2005). Nat. Mater..

[cit31] Collier C. P., Jeppesen J. O., Luo Y., Perkins J., Wong E. W., Heath J. R., Stoddart J. F. (2001). J. Am. Chem. Soc..

[cit32] Green J. E., Wook Choi J., Boukai A., Bunimovich Y., Johnston-Halperin E., DeIonno E., Luo Y., Sheriff B. A., Xu K., Shik Shin Y., Tseng H.-R., Stoddart J. F., Heath J. R. (2007). Nature.

[cit33] Collier C. P., Mattersteig G., Wong E. W., Luo Y., Beverly K., Sampaio J., Raymo F. M., Stoddart J. F., Heath J. R. (2000). Science.

[cit34] Gao S., Yi X., Shang J., Liu G., Li R.-W. (2019). Chem. Soc. Rev..

[cit35] Fung E. D., Gelbwaser D., Taylor J., Low J., Xia J., Davydenko I., Campos L. M., Marder S., Peskin U., Venkataraman L. (2019). Nano Lett..

[cit36] Li J., Zhang H. F., Shao G. Q., Wu B. L., Ouyang S. X. (2014). Europhys. Lett..

[cit37] Wang Y., Zhang Q., Astier H. P. A. G., Nickle C., Soni S., Alami F. A., Borrini A., Zhang Z., Honnigfort C., Braunschweig B., Leoncini A., Qi D.-C., Han Y., del Barco E., Thompson D., Nijhuis C. A. (2022). Nat. Mater..

[cit38] Cho B., Song S., Ji Y., Kim T.-W., Lee T. (2011). Adv. Funct. Mater..

[cit39] Wang R., Okajima T., Kitamura F., Matsumoto N., Thiemann T., Mataka S., Ohsaka T. (2003). J. Phys. Chem. B.

[cit40] Chiechi R. C., Weiss E. A., Dickey M. D., Whitesides G. M. (2008). Angew. Chem., Int. Ed..

[cit41] Cutinho J., Chang B. S., Oyola-Reynoso S., Chen J., Akhter S. S., Tevis I. D., Bello N. J., Martin A., Foster M. C., Thuo M. M. (2018). ACS Nano.

[cit42] Migliore A., Nitzan A. (2013). J. Am. Chem. Soc..

[cit43] Xu B., Dubi Y. (2015). J. Phys.: Condens. Matter.

[cit44] Sun W., Gao B., Chi M., Xia Q., Yang J. J., Qian H., Wu H. (2019). Nat. Commun..

[cit45] Gupta R., Fereiro J. A., Bayat A., Pritam A., Zharnikov M., Mondal P. C. (2023). Nat. Rev. Chem..

[cit46] Kumar S., Strachan J. P., Williams R. S. (2017). Nature.

[cit47] Kumar S., Williams R. S., Wang Z. (2020). Nature.

[cit48] Perrin M. L., Frisenda R., Koole M., Seldenthuis J. S., Gil J. A. C., Valkenier H., Hummelen J. C., Renaud N., Grozema F. C., Thijssen J. M., Dulić D., van der Zant H. S. J. (2014). Nat. Nanotechnol..

[cit49] Thompson D., Barco E. d, Nijhuis C. A. (2020). Appl. Phys. Lett..

[cit50] Jia C., Grace I. M., Wang P., Almeshal A., Huang Z., Wang Y., Chen P., Wang L., Zhou J., Feng Z., Zhao Z., Huang Y., Lambert C. J., Duan X. (2020). Chem.

[cit51] Kumar S., Merelli M., Danowski W., Rudolf P., Feringa B. L., Chiechi R. C. (2019). Adv. Mater..

[cit52] Darwish N., Aragonès A. C., Darwish T., Ciampi S., Díez-Pérez I. (2014). Nano Lett..

[cit53] Kim T.-W., Zeigler D. F., Acton O., Yip H.-L., Ma H., Jen A. K. Y. (2012). Adv. Mater..

[cit54] Ji Y., Zeigler D. F., Lee D. S., Choi H., Jen A. K. Y., Ko H. C., Kim T.-W. (2013). Nat. Commun..

[cit55] Cho B., Kim T.-W., Song S., Ji Y., Jo M., Hwang H., Jung G.-Y., Lee T. (2010). Adv. Mater..

[cit56] Pecqueur S., Mastropasqua Talamo M., Guérin D., Blanchard P., Roncali J., Vuillaume D., Alibart F. (2018). Adv. Electron. Mater..

[cit57] Li Z., Yu X. (2024). Neuromorphic Comput. Eng..

[cit58] Zhang Q., Wang Y., Nickle C., Zhang Z., Leoncini A., Qi D.-C., Sotthewes K., Borrini A., Zandvliet H. J. W., del Barco E., Thompson D., Nijhuis C. A. (2024). Nat. Commun..

[cit59] Lindsey J. S., Bocian D. F. (2011). Acc. Chem. Res..

[cit60] Zhu H., Li Q. (2017). Redox - Principles and Advanced Applications.

[cit61] Wang Z., Li Z., Li C., Ji X., Song X., Yu X., Wang L., Hu W. (2023). Proc. Natl. Acad. Sci. U. S. A..

[cit62] Singh A., Dahanayaka D. H., Biswas A., Bumm L. A., Halterman R. L. (2010). Langmuir.

[cit63] Chen X., Roemer M., Yuan L., Du W., Thompson D., del Barco E., Nijhuis C. A. (2017). Nat. Nanotechnol..

[cit64] Han Y., Maglione M. S., Diez Cabanes V., Casado-Montenegro J., Yu X., Karuppannan S. K., Zhang Z., Crivillers N., Mas-Torrent M., Rovira C., Cornil J., Veciana J., Nijhuis C. A. (2020). ACS Appl. Mater. Interfaces.

[cit65] Weiss E. A., Kaufman G. K., Kriebel J. K., Li Z., Schalek R., Whitesides G. M. (2007). Langmuir.

[cit66] Yuan L., Breuer R., Jiang L., Schmittel M., Nijhuis C. A. (2015). Nano Lett..

[cit67] Reus W. F., Nijhuis C. A., Barber J. R., Thuo M. M., Tricard S., Whitesides G. M. (2012). J. Phys. Chem. C.

[cit68] Garrigues A. R., Yuan L., Wang L., Mucciolo E. R., Thompon D., del Barco E., Nijhuis C. A. (2016). Sci. Rep..

