# Supplementary material for: Molecular-scale in-operando reconfigurable electronic hardware
Source: Nanoscale Horiz. 2024 Nov 27;10(2):349–58. doi: 10.1039/d4nh00211c (PMC11623307; doi:10.1039/d4nh00211c)
Supplement: NH-010-D4NH00211C-s001 [file NH-010-D4NH00211C-s001.pdf]

Supplementary Information (SI) for Nanoscale Horizons.
This journal is © The Royal Society of Chemistry 2024

Supplementary Information for

Molecular-Scale In-Operando Reconfigurable Electronic Hardware

Yulong Wang,[1†] Qian Zhang,[1,2†] Cameron Nickle,[3†] Ziyu Zhang,[1] Andrea Leoncini,[1] Dong-Chen Qi,[4] Alessandro Borrini,[5] Yingmei Han,[1] Enrique del Barco[3], Damien Thompson[6*], and Christian A. Nijhuis[5*]*

[1]Department of Chemistry, National University of Singapore, 3 Science Drive 3, Singapore 117543.

[2]School of Chemistry and Chemical Engineering, Chongqing University, Chongqing 400044, China.

[3]Department of Physics, University of Central Florida, Orlando, Florida 32816 - USA.

[4]Centre for Materials Science, School of Chemistry and Physics, Queensland University of Technology, Brisbane, Queensland 4001, Australia.

[5]Hybrid Materials for Opto-Electronics Group, Department of Molecules and Materials, MESA+ Institute for Nanotechnology, Molecules Center and Center for Brain-Inspired Nano Systems, Faculty of Science and Technology, University of Twente, 7500 AE Enschede, The Netherlands.

[6]Department of Physics, Bernal Institute, University of Limerick, Limerick V94 T9PX, Ireland.

[†]These authors contributed equally to this work

*Authors to whom correspondence should be addressed: chmnca@nus.edu.sg, damien.thompson@ul.ie and delbarco@ucf.edu.

S1. Materials, Methods and Synthesis of HATNA derivatives.

Sodium rhodizonate dibasic, 4-amino-3-nitrophenol, potassium thioacetate (KSAc), tin(II) chloride dihydrate ($SnCl_2 \cdot 2H_2O$), 1,2-phenylenediamine, 1,10-dibromodecane, conc. nitric acid (HNO_3, 65 %), potassium carbonate (K_2CO_3), conc. hydrochloric acid (HCl, 37 %), perchloric acid ($HClO_4$, 70%) and sodium hydroxide (NaOH) were purchased from Sigma-Aldrich, Alfa Aesar and Tokyo Chemical Industry (TCI) and used as received. Acetonitrile (MeCN) (Fulltime Chemical) and ethyl acetate (EA) (VWR Chemicals) were HPLC grade, while the absolute ethanol (EtOH), dichloromethane (DCM), dimethylformamide (DMF) and chloroform ($CHCl_3$) were analytical grade from VMR Chemicals also and used without further purification. THF (VWR Chemicals, HPLC) was distilled over sodium with benzophenone as indicator. Distilled water (18.2 MΩ cm) was supplied by a milli-Q system.

The SAMs were derived from a 5,6,11,12,17,18-hexaazatrinaphthylene (HATNA) thioacetate derivative that was synthesized and characterized as reported in our recent work.[1] All reactions were carried under an atmosphere of nitrogen. Scheme S1 shows the synthesis route of the HATNA derivative **5** used to form the SAMs. The synthesis route is the same as reported in our previous work where the SAMs were derived from the thiol-derivative.[1] Here, all SAMs were derived from **5** (instead of the thiol derivative used in our previous work) and therefore we provide a complete SAM characterization below. The advantage of using compound 5 instead of the thiol derivative is that 5 is stable whereas the thiol is prone to oxidation. For the sake of completion, we provide briefly the synthesis procedure below and refer to the full characterization and synthesis detail of compounds 1-5 to ref [1].

Compound quinoxalino[2,3-a]phenazine-6,7-dione (**1**) was synthesized from the equal stoichiometry of sodium rhodizonate dibasic and 1,2-phenylenediamine, and then the remaining solid was oxide with conc. HNO_3. The yellow crude product (**1**) was collected and used for the next step reactions directly.

Compound 4-((10-bromodecyl)oxy)benzene-1,2-diamine (**3**) was synthesized began from 4-amino-3-nitrophenol and excess 1,10-dibromodecane to obtain the product 4-((10-bromodecyl)oxy)-2-nitroaniline (**2**) with a long alkyl chain, and then the intermediate product (**2**) was further reduced with tin dichloride at the acid condition to obtain the diamine product (**3**), which was also used directly for the next step without further purification.

For the HATNA thioacetate compound (**5**), it was synthesized started from the dione compound (**1**) and diamine compound (**3**), and obtained the HATNA compound (**4**) with an alkyl chain bromide as the terminal. Then the bromide HATNA derivative was reacted with potassium thioacetate to obtain the final product HATNA thioacetate (**5**).

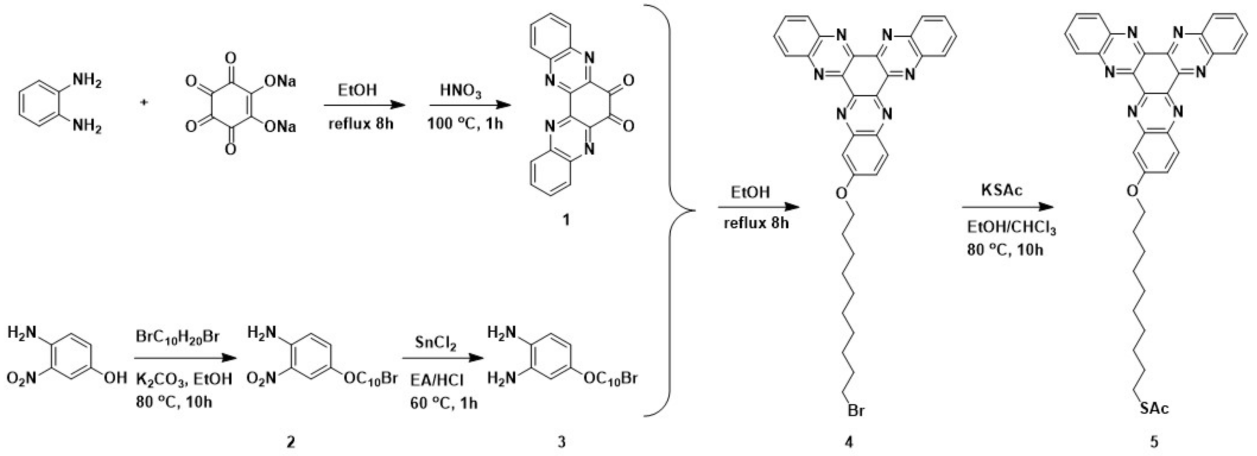

Scheme S1. Synthesis route of the target HATNA derivative **5**.

S2. Self-assembled monolayer (SAM) formation.

The HATNA SAMs were prepared following an established method.[2] We dissolved 1.0 mg AcS-C_{10}-HATNA in 5 ml freshly distilled tetrahydrofuran (THF, with a concentration of 0.33 M), then the solution was flushed with N_2 for about 15 min to remove the oxygen. After that, 20μl ammonia (26-28%) was added, and freshly prepared Au surfaces were immersed in the solution. After about 24 h, the Au substrates were taken out from the solution and washed immediately with plenty of THF and ethanol, and then the substrates were gently dried with N_2 flow.

S3. Electrode preparation.

The top electrode was prepared with the well-known EGaIn technique. Here, an EGaIn alloy to form a cone shape tip is used which is then used to form contacts with the SAMs as described in detail in previous reports.[3] The bottom electrode was fabricated with the well-known template-stripped method.[4] First, a 200 nm thick Au (99.999% Au granules, ACI Alloys) thin film was deposited on a Si wafer (with a native SiO_2 thin layer on surface, Syst Integration Pte Ltd) using a thermal evaporator (Shen Yang Ke Yi), then pre-cleaned glass slides were adhesive on the Au surface with thermal glue (EPOTEK 353ND). After that, the Au thin film was heated at 80 °C for 3 hours to cure the adhesive. The Au electrode was stored in a dry box and template-stripped immediately before use.

S4. Electrochemistry.

The cyclic voltammetry measurements of the HATNA SAMs were conducted with an Autolab PGSTAT302T setup equipped with NOVA 1.10 software. In the measurements, a Pt plate was worked as the counter electrode, an Ag/AgCl electrode was

used as the reference electrode, the Au substrate coating with SAM was supported as the work electrode, and A $HClO_4$ aqueous solution (2M) was used as the electrolyte. We recorded the results between -0.4 V and 1.0 V with various scan rates from 0.02 V/s to 100 V/s.

The HATNA SAMs on the Au were derived from compound 5 and measured with cyclic voltammetry (Fig. S1). The cyclic voltammograms show four redox waves, which is similar to that for HATNA SAMs derived from the respective thiol as in our previous report,[1] indicating a similar redox process involved in six successive proton-coupled electron transfer (PCET) steps. Peak 1 is associated with the initial protonation processes, which is irreversible as shown in the Fig. S1. The surface coverage of the HATNA SAM (Γ_{HATNA}) was calculated with equation S1. The value of Γ_{HATNA} is $2.73 \pm 0.35 \times 10^{-10}$ mol/cm^2, which was calculated at a scan rate of 1.0 V/s by integrating the main peaks P2, P3 and P4 (see Fig. S1) and taking electrons transferred number n = 4, as these redox waves were overlapped with each other (i.e., using the same approach as in ref 1); The value of Γ_{HATNA} is close to our previous report of 2.5×10^{-10} for the HATNA thiol SAMs and theoretical value of 2.8×10^{-10} mol/cm^2 with a tilt angle of 32°.

$$\Gamma_{\mathrm{HATNA}} = Q_{\mathrm{tot}}/nFA \qquad \text{(S1)}$$

where Γ_{HATNA} is the surface coverage of HATNA SAM (mol/cm^2), Q_{tot} is the total charge integrated from the redox wave, n is the number of electrons transferred in the redox reaction, F is the Faraday constant (96485 C/mol) and A is the contact area of the working electrode with the electrolyte (0.36 cm^2).

The energy levels of the HATNA SAM were also studied using CV measurements. The LUMO level (E_{LUMO}) of the HATNA SAM was accounted with the equation S2, with

the value about -4.09 eV. To further probe the energy gap (E_{gap}) between the HOMO and LUMO levels obtained from the UV measurement in our previous report,[1] the HOMO level (E_{HOMO}) was also calculated with the equation $E_{LUMO} = E_{HOMO} + E_{gap}$.

$$E_{LUMO} = E_{abs,\ NHE} - eE_{1/2,NHE} \qquad (S2)$$

wherein, $E_{abs,\ NHE}$, equals to -4.5 eV, is the absolute potential energy of the normal hydrogen electrode, e is the charge of one electron (1.602×10^{-19} C), and $E_{1/2,NHE}$ is the formal half-wave potential versus normal hydrogen electrode (NHE). The absolute potential energy of Ag/AgCl is +0.197 V vs. NHE.

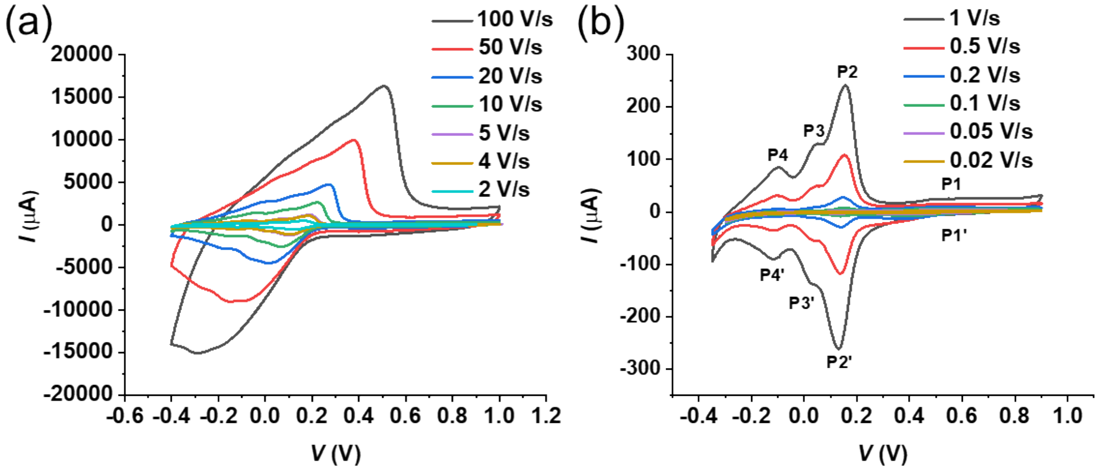

Fig. S1. Cyclic voltammograms of a S-C_{10}-HATNA SAM on Au in 2 M aqueous $HClO_4$ recorded with scan rates in the ranges of 1-10 V/s (a) and 0.05-0.5 V/s (b).

S5 Surface characterization.

The surface characterizations of the HATNA SAMs were performed with X-ray photoelectron spectroscopy (XPS) and ultraviolet photoelectron spectroscopy (UPS), and near-edge X-ray absorption fine structure (NEXAFS) spectroscopy, which were supported by the Surface, Interface and Nanostructure Science (SINS) beamline of the Singapore

Synchrotron Light Source (SSLS). The measurements were conducted on template-stripped Au surfaces coated with S-C_{10}-HATNA SAM, with similar procedures in our previous work.[5] In the high-resolution XPS measurements, the photon energy was optimized to obtain the optimal signals according to different elements, with the value at 350 eV for S 2*p* and C 1*s*, 600 eV for N 1*s* and 650 eV for O 1*s* spectra. All the XPS measurements were recorded at two different photoelectron take-off angles with values of 90° (normal emission, NE) and 40° (grazing emission, GE). By removing the background with the Shirley method, the XPS peaks were fitted with a fixed ratio of Gaussian (70%) and Lorentzian (30%) based on the *pseudo*-Voigt functions.

The XPS, UPS and NEXAFS measurements have been recorded for the HATNA SAMs and are shown in Figs. S2-S3. For the S 2*p* spectra of the Au-S-C_{10}-HATNA SAMs, only one doublet peak was observed at ~162.0 eV, which is also similar to the value of chemical bonding of sulfur to the Au surface as previously reported,[1] indicating that a high-quality SAM was fabricated also with the HATNA thioacetate precursor. The C 1*s* spectra show two sets of singlet peaks at ~284.8 eV and ~286.0 eV, which corresponds to the carbon in the alkyl chain (with the C-C bonds and C-S bond), and the carbon in or close to the HATNA core (with the C-N bond and C-O bond), separately. The N 1*s* spectra show only a singlet peak at 399.2 eV since all the N atoms in the HATNA core have a similar chemical environment. In the O 1s spectra, two singlet peaks were identified at ~533.2 eV and ~532.1 eV. The main peak at ~533.2 eV corresponds to the O atom close to the HATNA core with a C-O-C bond. The small peak at ~532.1 eV is most likely attributable to the physical-absorbed contaminations from the environment. The thickness of the HATNA SAM (d_{SAM}) was about 23.6±1 Å, which was calculated with the peak intensities

of S 2*p* peaks at about 162.0 eV measured with the incident angle at 90° and 40° separately, using the same method as described in detail in ref [3].

The carbon K-edge NEXAFS spectra (Fig. S3a) show three sharp peaks at 283.9 eV, 285.0 eV, and 285.7, which are attributed to the electron transitions in N atoms to the π^* orbitals for the LUMO, LUMO+1 and LUMO+2 levels, respectively. The peak at 287.0 eV can be assigned to π electrons delocalized over the whole HATNA core that are excited to the LUMO+3 level. The peak analysis is in agreement with the DFT calculations in our previous report.[1]

We determined the energy of the HOMO level of the HATNA SAMs using the method described in our previous reports.[1, 3] The HOMO onsets (δE_{HOMO}) were determined from the valence band spectra (Fig. S3b), and the work function (Φ) of the HATNA SAMs were obtained from the secondary electron cut-off (SECO) spectra (Fig. S3c). The HOMO level was calculated with equation $E_{HOMO} = -(\delta E_{HOMO} + \Phi)$, which was similar to the results obtained from the CV measurements and DFT calculations, as listed in Table S1. the energy of the LUMO level was estimated using equation $E_{LUMO} = E_{HOMO} + E_{gap}$, where E_{gap} was determined from the UV spectrum in our previous report.[1]

Table S1. Summary of surface characterization of HATNA molecules on Au

	Energy levels (eV)						Γ^c (10^{-10} mol/cm^2)	d_{SAM} (Å)
	E_{HOMO}	E_{LUMO}	E_{LUMO+1}	E_{LUMO+2}	E_F[a]	E_{gap}[b]		
**Exp.**	-6.90[d]	-4.09[d]				2.81	2.73±0.35	23.6±1.0[e]
	-6.28[f]	-3.47[f]			4.18			
**Theor.**[g]	-6.17	-2.79	-2.37	-2.31				

[a] Measured with UPS.
[b] Measured with UV.
[c] Measured with SAM CV.
[d] Measured with SAM CV and UV
[e] Measured with XPS
[f] Measured with UPS and UV.
[g] Calculated with DFT.

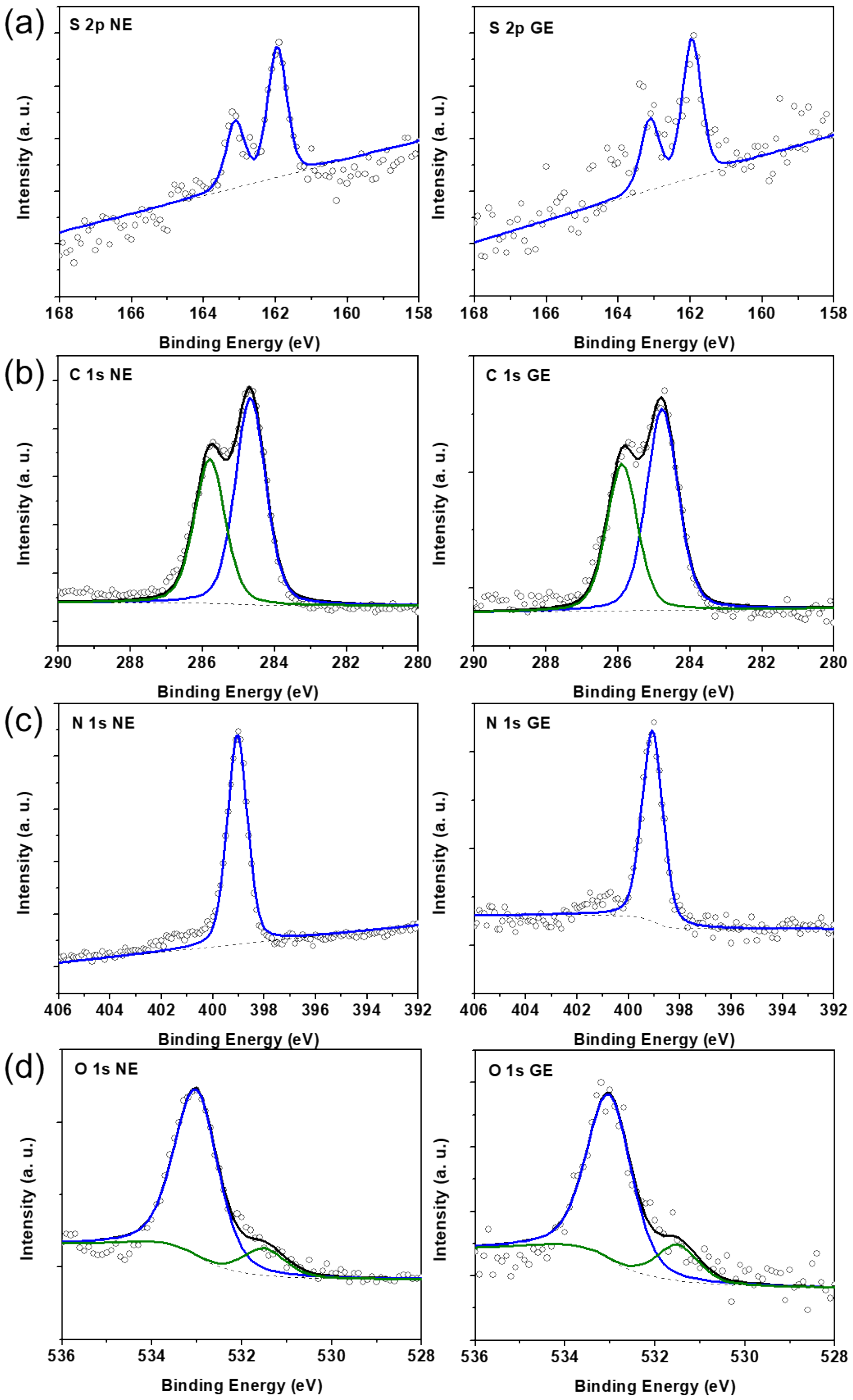

Fig. S2. High resolution XPS spectra of (a) S 2*p*, (b) C 1*s*, (c) N 1*s*, and (d) O 1*s* recorded from a SAM of Au-S-C_{10}-HATNA.

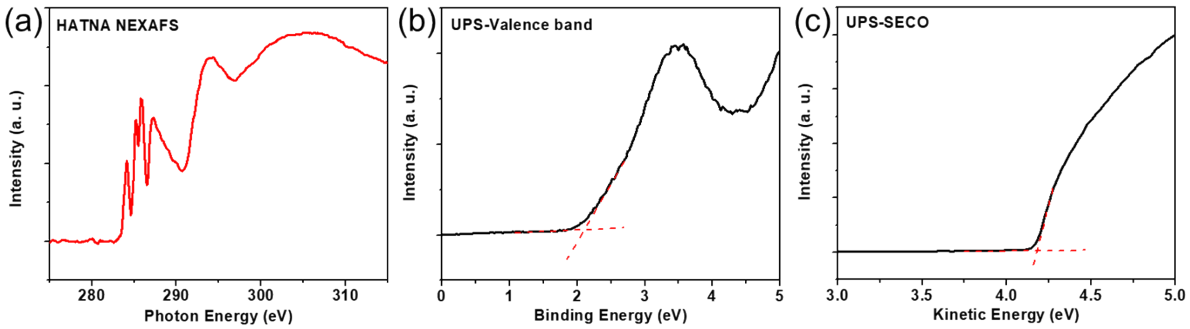

Fig. S3. (a) C K-edge NEXAFS, (b) valence band and (c) SECO spectra recorded from a SAM of Au-S-C_{10}-HATNA.

S6. Junction fabrication and statistical analysis.

S6.1 Junction fabrication and electrical measurements in air.

The junctions were fabricated with a grounding Au bottom electrode, a HATNA monolayer, and a cone-shaped GaOx/EGaIn top electrode, in which the bias was applied on the top EGaIn electrode (Fig. S4), similar to the previous report.[3] The electrical measurements were carried out with a LabVIEW-controlled Keithley 6430 source meter. We recorded the J(V) curves with voltage from 0 V→ +1 V → 0 V → -2 V → 0 V with every 20 mV in step.

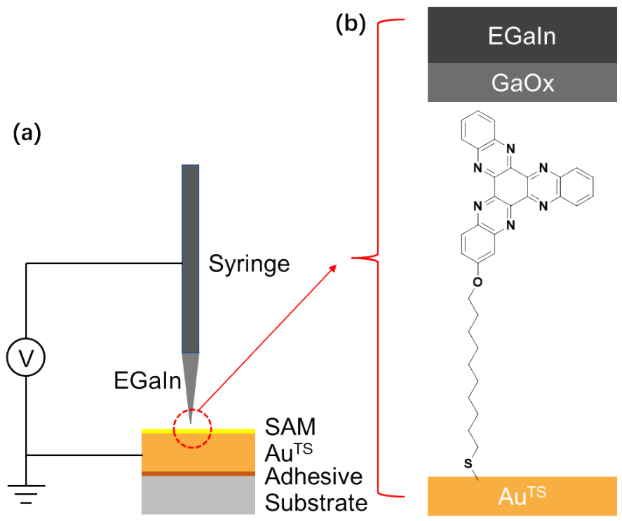

Fig. S4. Schematic illustration of the HATNA based molecular junction structure which was constructed using the well-established EGaIn technique. For details please refer to the methods section and previous reports.[6, 7] (a) The HATNA SAM was supported by a template-stripped Au bottom electrode (Au^{TS}). Using a Hamilton syringe (mounted on a micromanipulator) filled with EGaIn, a cone-shaped tip of EGaIn was formed. This EGaIn tip was then brought into contact with the HATNA monolayer to complete the junctions. We applied the bias voltage to the cone-shaped GaO_x/EGaIn tip using a LabVIEW-controlled Keithley 6430 source meter, and the Au electrode was grounded. (b) Zoom-in to highlight the HATNA molecular junction.

S6.2 Statistical analysis of *J*(V) characteristics.

The statistical analysis of *J*(V) data was performed with the median averages of the current ($<\log_{10}|J|>_m$) and the median absolute deviations (σ_m), as recommended in the previous report,[8] because this method does not reply on presumptions regarding the type of data distribution as explained in more detail in ref [1]. The values of $<\log_{10}|J|>_m$ and σ_m were calculated from the $\log_{10}|J|$ values measured at each bias step. The heatmaps of the *J*(V)

curves were created using OriginPro 2019b software with 2D kernel density estimations. In the 2D kernel density estimations, the density values were calculated based on a bi-dimensional Gaussian kernel, and the bandwidth was selected through a bivariate kernel density estimator with a grid size of 100.

The *J*(V) measurements of the HATNA junctions were conducted with the junction structure Au^{TS}-S-C_{10}-HATNA//GaO_x/EGaIn. We repeated the measurements for three separate Au substrates, with about 20 junctions for each Au substrate. For each junction, only three traces of *J*(V) curves were recorded. During the *J*(V) measurements, some junctions were unstable or shorted with the current increasing sharply or out of the compliance limit of the electrometer, which were excluded from the statistical analysis. We performed the statistical analysis of *J*(V) characteristics with the median averages of the current ($<\log_{10}|J|>_m$) and the median absolute deviations (σ_m), because this method can minimize the influence of the data distribution.[8] The heatmap of the *J*(V) measurements was created with OriginPro 2019b software using 2D kernel density estimations, see the detailed description in our previous report.[1]

Table S2. The *J*(V) and NDR statistics for the Au^{TS}-S-C_{10}-HATNA//GaO_x/EGaIn junctions measured at a scan rate of 10 mV/s.

Molecule	Total junctions	Working junctions	Traces	Yield (%)	R_{PtV}[a]	$R_{on/off}$[a]
AcS-C_{10}-HATNA	63	57	171	90.5	14.8±2.8	280±115

[a] log-median values; error is the mean absolute deviation.

S6.3 Statistical analysis.

We have studied the electric characterization of the HATNA SAMs with *J*-*V* measurements for a total of 63 junctions with a high yield of up to 90.5%. The statistical results are

summarized in Table S2 and the $<\log_{10}|J|>_m$ *vs.* V curves along with σ_m are shown in Fig. S5. For each single J(V) trace, the peak-to-valley ratio (R_{PtV}) was calculated with the ratio of current density between the NDR peak and valley. The on/off ratio ($R_{on/off}$) was determined as the ratio of current density on the NDR peak and the current at the forward bias of the NDR peak. The R_{PtV} and $R_{on/off}$ values calculated from all traces were analyzed with the median method, as described in our previous report,[1] with the median averages ($<R_{PtV}>_m$ and $<R_{on/off}>_m$) and the log-median absolute deviations ($\sigma_{PtV,m}$ and $\sigma_{on/off,m}$) as listed in Table S2 and along with the histogram of the R_{PtV} and $R_{on/off}$ distributions illustrated in Fig. S5.

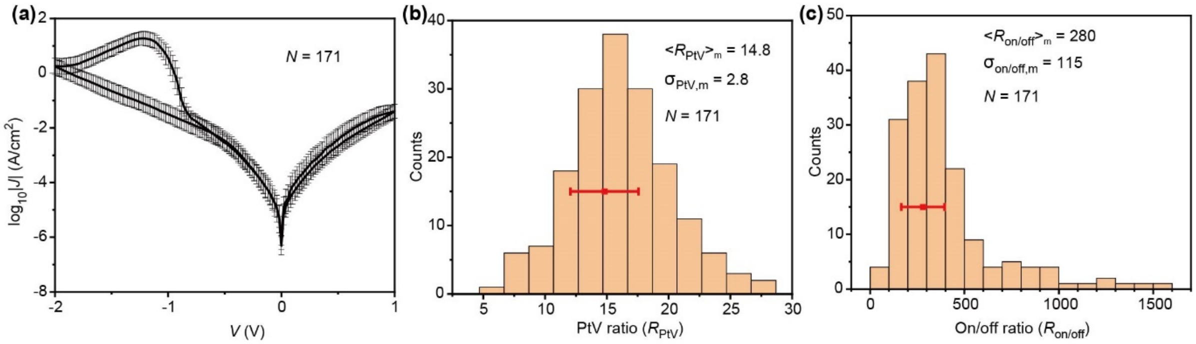

Fig. S5. Electrical characteristics of the Au^{TS}-S-C_{10}-HATNA//Ga_2O_3/EGaIn junctions in air. (a) $<\log_{10}|J|>_m$ *vs.* V; the error bars are σ_m. (b) Histogram of R_{PtV} (red circle and error bars indicate $<R_{PtV}>_m$ and $\sigma_{PtV,m}$). (c) Histogram of $R_{on/off}$ (red circle and error bars indicate $<R_{on/off}>_m$ and $\sigma_{on/off,m}$).

S6.4 Bias window dependent measurements.

We measured the junctions at different bias window at scan rate of 10 mV/s, including symmetric voltage scan, positive voltage dependent measurement and negative voltage dependent measurement (as shown in Fig. 3 and Figs. S6-S10). For the symmetric bias window dependent measurement, we measure the J(V) curves at ± 0.25 V, ± 0.50 V,

±0.75 V, ±1.0 V, ±1.25 V, ±1.50 V and ±1.75 V. For the positive voltage dependent measurement, we control the negative at -2.0 V (or at -1.2 V), and change the positive voltage at 0 V, 0.25 V 0.50 V, 0.75 V, 1.0 V, 1.25 V, 1.50 V and 1.75 V. In the negative voltage dependent measurement, we set the positive voltage at 1.0 V, and adjust the negative voltage at 0 V, -0.25 V, -0.50 V, -0.75 V, -1.0 V, -1.25 V, -1.50 V, -1.75 V and -2.0 V. For the reversible reconfigurability experiment, we measure the *J*(V) curves from ±0.50 V, ±0.75 V, ±1.0 V, ±1.25 V, ±1.50 V to ±1.75 V, and then scan backward with the same voltage windows in reverse order. Or keep the positive voltage at +1.0 V, and increase the negative from 0 V, -0.25 V, -0.50 V, -0.75 V, -1.0 V, -1.25 V, -1.50 V, -1.75 V to -2.0 V and then scan backward with the same voltage windows in reverse order (as shown in Fig. 5 and Fig. S11).

The reconfigurable feature of our junctions was measured by changing the applied bias window symmetrically (Figs. S6 and S7), or by keeping the maximum applied voltage at -1.2 V (Fig. S10) or -2.0 V (Fig. S9). For the sake of completion, we also kept the positive applied voltage at 1.0 V while varying the negative applied voltage (Fig. S8). We measured 3 junctions for each kind of measurements to demonstrate the reproducibility of our system. All measurements were conducted at a scan rate of 10 mV/s. As discussed in the main text, typical off-resonant tunneling is observed at low bias (Fig. S12), but the junctions turn on and hysteretic behavior is observed at intermediated applied bias. At large negative bias, the junctions switch off again resulting in NDR. As a result, we can access two kinds of hysteretic behavior associated with normal memristive behavior and negative memristive behavior. From the positive voltage dependent measurements while keeping the negative bias at -2.0 V (Fig. S9), NDR can only be observed during the first scan but the NDR is

absent during the second scan when keeping the positive voltage at 0 V. The NDR effect can be restored with increasing positive from 0 V to 1.0 V, after that the NDR decreases gradually again at large positive voltages (V = 1.5 V and 1.75 V). While keeping the negative bias at -1.20 V (Fig. S10), we can achieve a large normal memristive behavior, with the highest on/off ratio up to 2.5×10^3 at V = -0.56 V because the H_n-HATNA moieties are not fully reduced (n varies between 0-2) during these experiments. At very large positive bias (1.5 V or 1.75 V; Figs. S6, S9 and S10), a small NDR feature appears which indicates that at large positive bias the LUMO or intergap state also enters the conduction window (red arrows in Fig. S6) and the H_n-HATNA can be reduced (partially) again off-setting the reset process resulting in a reduction of the turn on current and NDR feature.

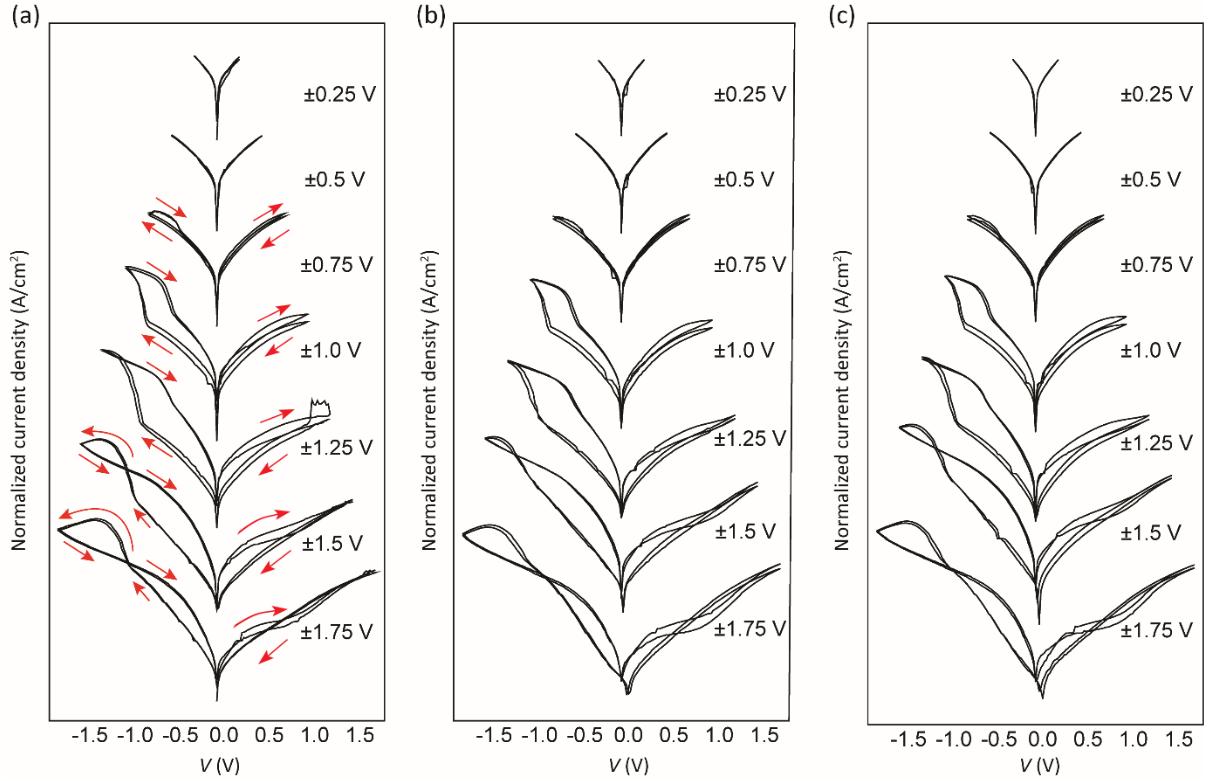

Fig. S6. Normalized semi-log plots of the J(V) curves recorded with the following bias windows: ±0.25 V, ±0.5 V, ±0.75 V, ±1.0 V, ±1.25 V, ±1.5 V and ±1.75 V. The panels

represent data obtained from three different junctions: (a) data set obtained from junction 1, (b) data set obtained from junction 2 (shown in the main text), and (c) data set obtained from junction 3.

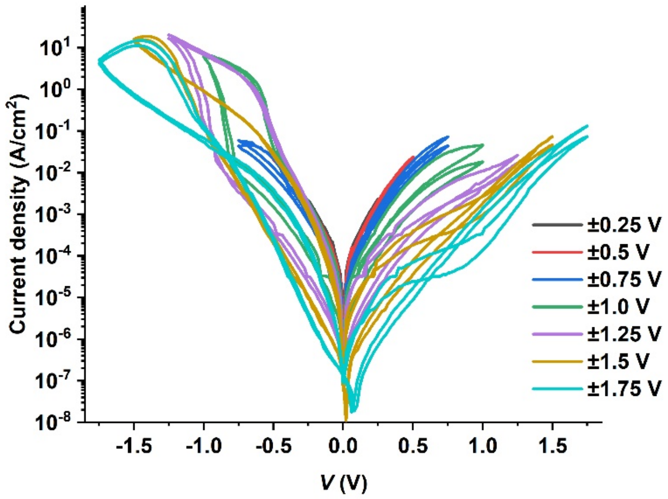

Fig. S7. The *J*(V) curves of junction 2 in Fig. S6b displayed following consistent scales, also as a comparison with normalized current density shown in the main text Fig. 3a.

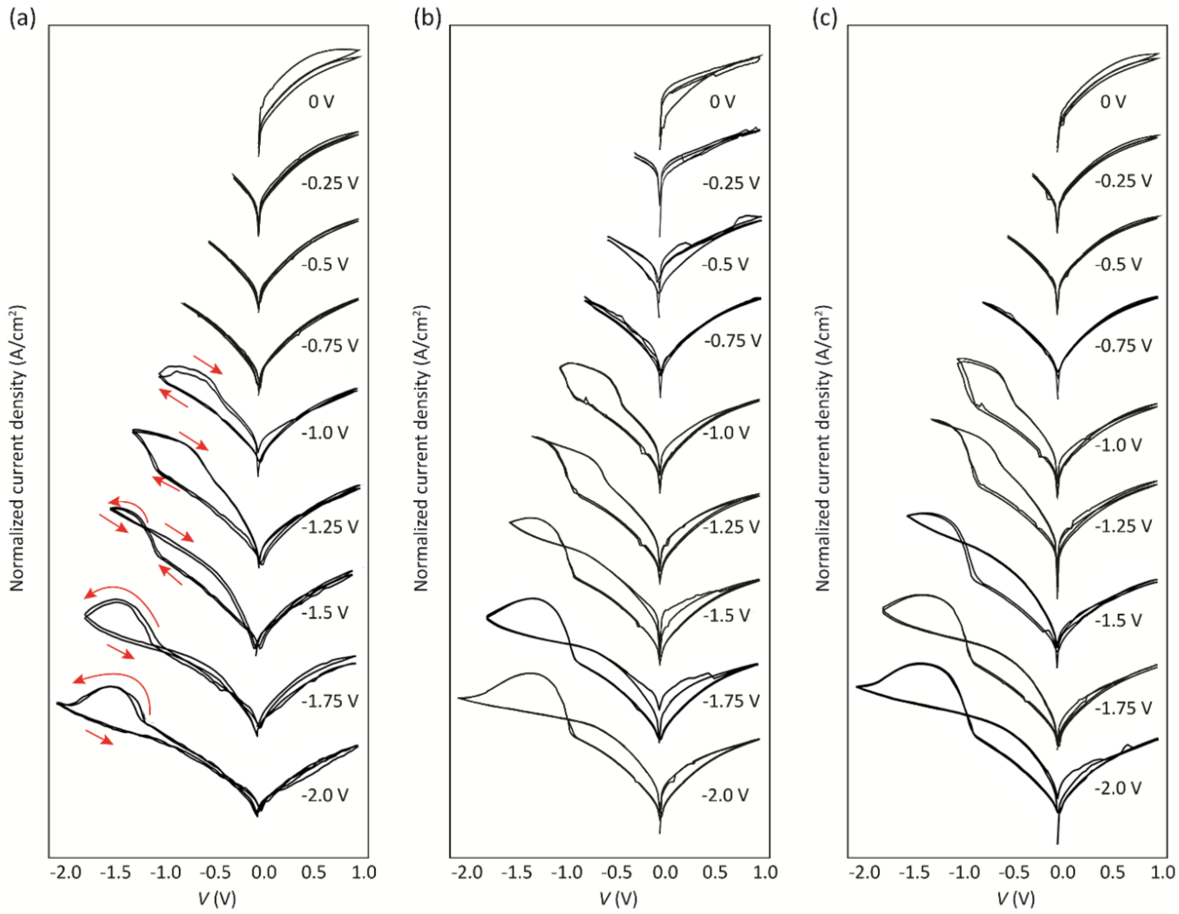

Fig. S8. Normalized semi-log plots of the *J*(V) curves recorded with maximum applied positive bias of 1.0 V and varying negative voltage from 0 to -2.0 V with intervals of 0.25 V. The panels represent data obtained from three different junctions: (a) data set obtained from junction 1 (shown in the main text), (b) data set obtained from junction 2, and (c) data set obtained from junction 3.

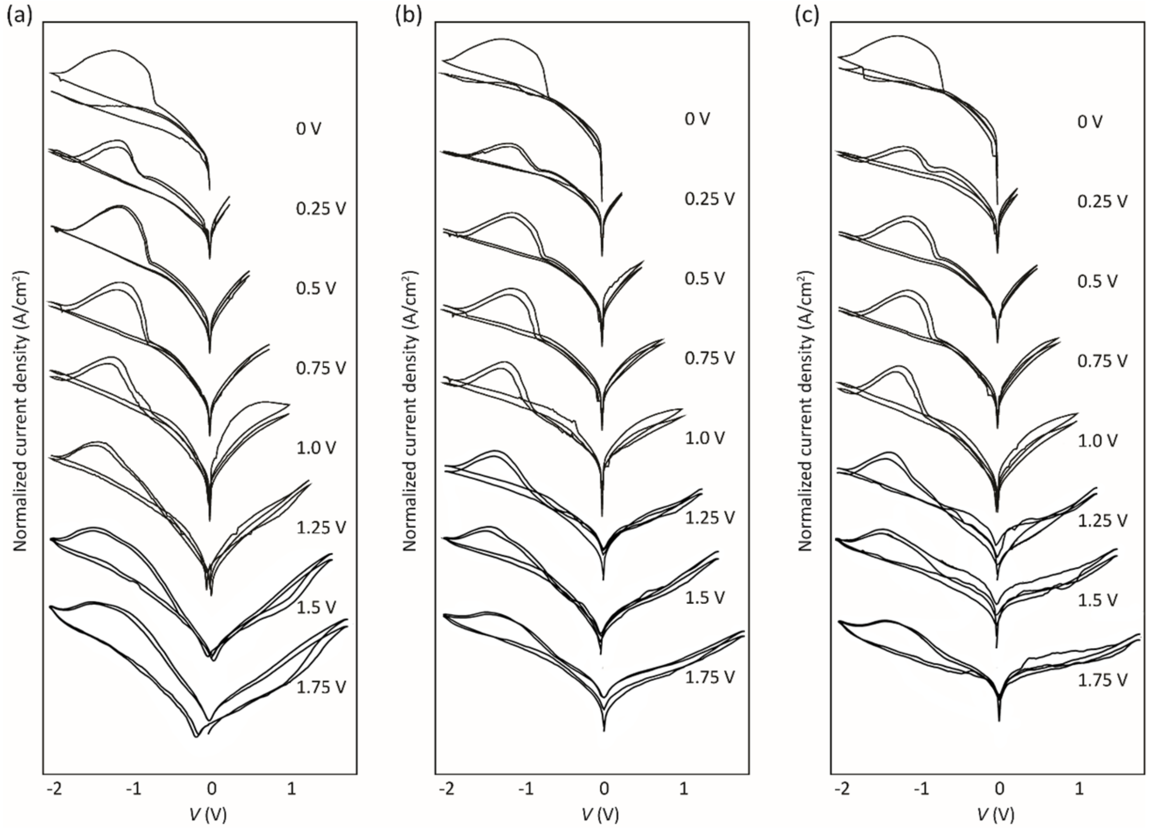

Fig. S9. Normalized semi-log plots of the *J*(V) curves recorded with ta maximum applied negative voltage of -2.0 V and varying positive voltage from 0 to 1.75 V in steps of 0.25 V. The panels represent data obtained from three different junctions: (a) data set obtained from junction 1, (b) data set obtained from junction 2 (shown in the main text), and (c) data set obtained from junction 3.

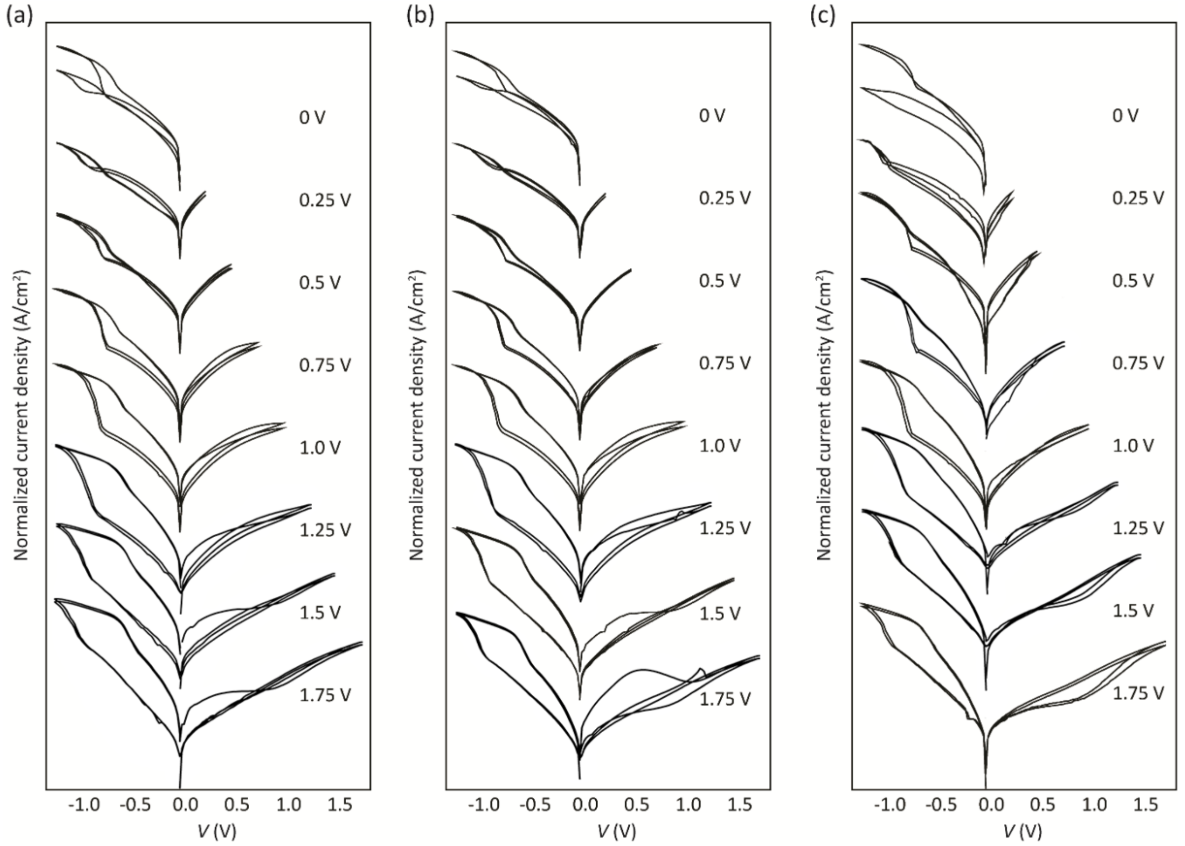

Fig. S10. Normalized semi-log plots of the *J*(V) curves recorded with a maximum applied negative voltage of -1.20 V and varying positive voltage of 0 to 1.75 V with intervals of 0.25 V. The panels represent data obtained from three different junctions: (a) data set obtained from junction 1 (shown in the main text), (b) data set obtained from junction 2, and (c) data set obtained from junction 3.

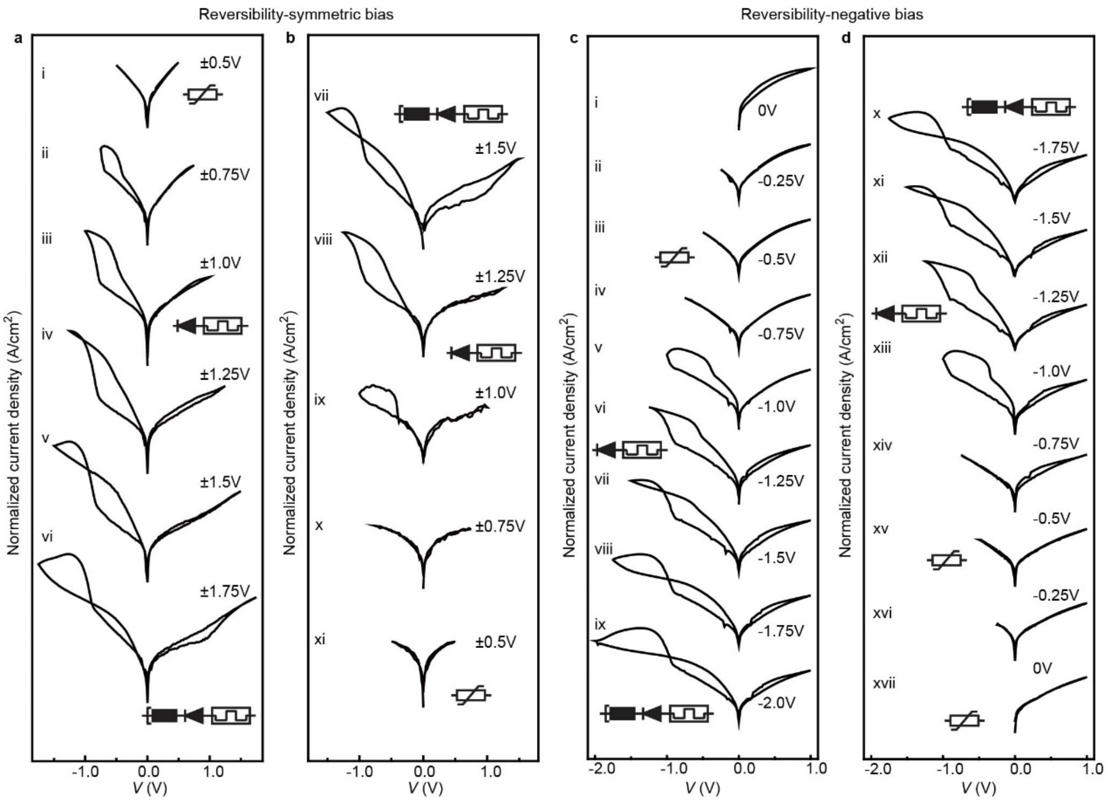

Fig. S11. Demonstration of reversible reconfiguration of the junctions. (**a-b**) with symmetric voltage window: the symmetric voltage window increased in (**a**) from 0.5 V to 1.75V, and then in (**b**) back to 0.5V. (**c-d**) with negative voltage dependent measurements: keeping the positive voltage at 1.0 V and the negative voltage in (**c**) changing from 0 V to -2.0 V and then in (**d**) back to 0 V. The same as the junction shown in Figs. 3 and 4, with increasing applied bias window the junction is reconfigured and the entire process is reversible with decreasing applied bias window and the junction can be reconfigured back to its original state.

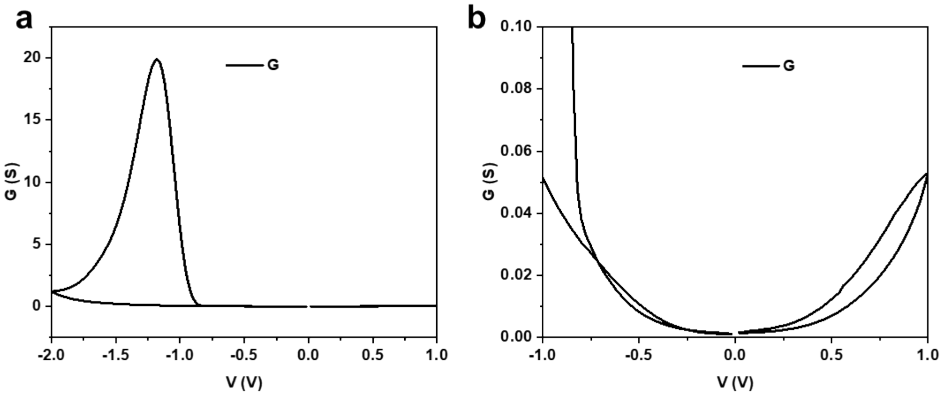

Fig. S12. The G-V curve for the HATNA junction. (a) with a voltage from +1 V to -2 V, and (b) with a voltage from +1 V to -1 V to highlight the non-linear behavior. The exponential increase of current with bias is characteristic of coherent tunneling meaning that the resistance of the junction is bias dependent. Hence the term variable resistor.

S7 Theoretical Modelling of the Transport Dynamics

S7.1 Description of the mechanistic modelling.

We analyzed the reconfigurable behavior of the junctions using a theoretical model developed by Migliore and Nitzan,[9] as described in our previous report.[1] According to the DFT calculations, the HATNA junctions can be configured into differing conduction modes (Fig. 2b-d). By choosing the bias window in which the HATNA junctions are operated, the junctions can be stabilized into providing different functionalities, including VR, D, MR, and NDR.

To understand this behavior, the current across the junction can be considered to have contributions from both the on-state and off state, with a given dynamical probability of being in one state or the other. Migliore and Nitzan describe this in their model which is reproduced in eq S3 where the off and on states are represented as (P^{AB}) and ($P^{\overline{AB}}$),

$$I(V) = P^{AB}(V)I^{AB}(V) + P^{\overline{AB}}(V)I^{\overline{AB}}(V) \qquad (S3)$$

where the probability of being in the on state can be related to that of the off state by $P^{AB}(V) = 1 - P^{\overline{AB}}(V)$. The currents, I^{AB} and $I^{\overline{AB}}$ represent the electrical transport through the on and off conduction states, respectively. I^{AB} and $I^{\overline{AB}}$ are described by the standard Landauer single-level tunnelling model[23] which is further defined in eq S6.

The probability of being in the on/off state is dynamic as it changes with subsequent PC-EECT reduction steps, the bias sweep rate, and the bias window. The probability is described by eq S4.

$$\frac{dP^{AB}}{dt} = \left(1 - P^{AB}\right) < R_{+PC} > - P^{AB} < R_{-PC} > \qquad (S4)$$

where $< R_{+PC} >$ and $< R_{-PC} >$ represent the rate of switching from the off to the on state and from the on to the off state, respectively. These rates of change are modelled using Marcus charge transfer theory as described by eq S5:

$$R_{+PC} = \frac{\gamma}{2}\sqrt{\frac{\pi k_B T}{\lambda}} exp\left[-\frac{\left(\alpha_{+PC} + \lambda\right)^2}{4\lambda k_B T}\right] \quad R_{-PC} = \frac{\gamma}{2}\sqrt{\frac{\pi k_B T}{\lambda}} exp\left[-\frac{\left(\alpha_{-PC} - \lambda\right)^2}{4\lambda k_B T}\right] \qquad (S5)$$

here λ is the reorganization energy associated with protonation, γ is the molecule-surrounding coupling parameter modulating the protonation process, and $\alpha_{ij} = \mu - E_{PC} + eV$, with E_{PC} representing an energy level associated to the reduction process, and μ is the electrochemical potential of the leads.

S7.2 Landauer formalism to account for the tunneling in the on and off states.

As stated previously, the currents in Migliori and Nitzan's model, eq S3, are represented by eq S6, with I^{AB} and $I^{\overline{AB}}$ accounting for the on and off states, respectively. These equations effectively model the current across the junction including the positions of the LUMO before (δE^{AB}) and after ($\delta E^{\overline{AB}} = \delta E^{AB} + \chi$) reduction ($\chi$ is defined as the

LUMO level difference before and after the reduction). Further, reduction of the molecule leads to a change in the coupling with the electrodes. The two tunneling rates Γ^{AB} and $\Gamma^{\overline{AB}} = \kappa\Gamma^{AB}$, defined as $\Gamma^{i} = \gamma^{L,i}\gamma^{R,i}/(\gamma^{L,i} + \gamma^{R,i})$ represent the rates before and after reduction with κ indicating the ratio of the between the on and off tunneling rates.

$$I^{AB,\overline{AB}} = \frac{nq}{2\pi\hbar}\int\int_{-\infty}^{\infty} dEdE' \, D_{E'}^{AB,\overline{AB}}(E)G_{\epsilon}^{AB,\overline{AB}}(E')\frac{\gamma_{L}^{AB,\overline{AB}}\gamma_{R}^{AB,\overline{AB}}}{\gamma_{L}^{AB,\overline{AB}} + \gamma_{R}^{AB,\overline{AB}}}[f_{L}(E) - f_{R}(E)]\#(S6)$$

where, n is the number of molecules in the junction, q is the electron charge, and $\hbar$ is the reduced Plank's constant. $D_{E'}^{np,p}(E)$ represents the density of states given by a Lorentzian function as shown in eq S7:

$$D_{E'}^{AB,\overline{AB}}(E) = \frac{\gamma^{AB,\overline{AB}}/2\pi}{\left(E - \left(E' + \left(\eta^{AB,\overline{AB}} - \frac{1}{2}\right)V\right)\right)^{2} + \left(\gamma^{AB,\overline{AB}}/2\right)^{2}}\#(S7)$$

where $\gamma^{AB,\overline{AB}} = \gamma_{L}^{AB,\overline{AB}} + \gamma_{R}^{AB,\overline{AB}}$ is the molecular level width determined by the tunneling rates between the molecule and the left (L) and right (R) electrodes, V is the bias voltage applied to the junction. The voltage division parameter $\eta^{AB,\overline{AB}}$ accounts for the asymmetry of the junction and the associated voltage drop between the HATNA moiety and the electrodes.

$G_{\epsilon}^{AB,\overline{AB}}(E')$ is a Gaussian distribution used to describe the dispersion of the molecular orbital energy of the on and off states in the large-area HATNA molecular tunneling junctions, as shown in eq S8:

$$G_{\epsilon}^{AB,\overline{AB}}(E') = A\,exp\left(\frac{\left(E' - \varepsilon^{AB,\overline{AB}}\right)^{2}}{2\left(\sigma^{AB,\overline{AB}}\right)^{2}}\right)\#(S8)$$

where, $\varepsilon^{AB,\overline{AB}}$ is the zero-bias energy offset of the molecular orbitals with respect to the electrochemical potential of the electrodes, and $\sigma^{AB,\overline{AB}}$ represents its energy dispersion (width of the distribution).

$f_{L/R}(E)$ expresses the Fermi occupation distributions for each of the electrodes, which is generated from the thermal broadening of the electrodes, as shown in eq S9:

$$f_{L/R}(E) = \frac{1}{1 + e^{\frac{(E \pm V/2)}{k_B T}}} \#(S9)$$

S7.3 Fitting of the data.

We analyzed the switching behavior using the model described in the two earlier sections. Both calculated and experimental data are shown in Fig. 3. Additional measured datasets and the corresponding calculated curves, are shown in Fig. S13.

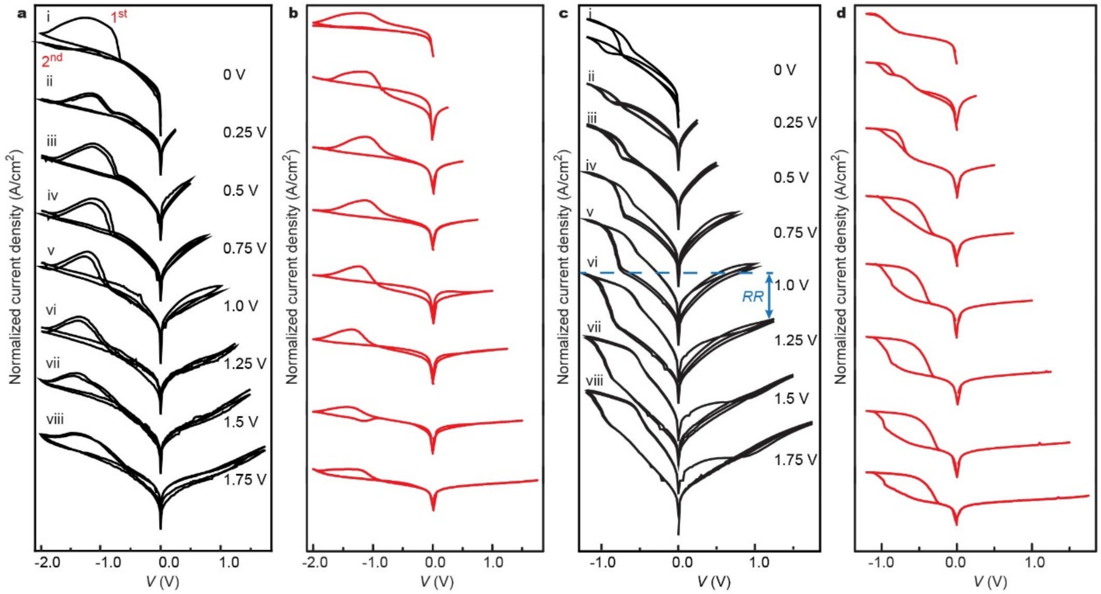

Fig. S13. Reconfigurable behavior of the junctions. (a) Positive voltage dependent measurement keeping maximum negative bias at -2.0 V. 1st and 2nd labels denote the 1st and 2nd voltage sweeps. (**b**) Calculated *I*-*V* cycles (red) for asymmetric positive bias cycles with fixed negative voltages -2 V. (**c**) Positive voltage dependent measurement keeping

maximum negative bias at -1.2 V. (**d**) Calculated *I*-*V* cycles (red) for asymmetric positive bias cycles with fixed negative voltages -1.2 V.

A total of 32 different data sets were fitted using the above model. Given that each data set represented the current profile of the same molecular species, tight constraints were placed on the modelling. Experimentally obtained values such as the temperature (T=300K) and the number of molecules (n = 300, which is very close to an experimentally determined number reported in ref [3]) were fixed for all data sets. Further, the asymmetry parameters η^{AB} and $\eta^{\overline{AB}}$ and dispersion parameters σ^{AB} and $\sigma^{\overline{AB}}$ were also fixed for all curves with $\eta^{AB} = \eta^{\overline{AB}} = 0.67$ and $\sigma^{AB} = \sigma^{\overline{AB}} = 76$ meV, respectively. These values fall within the range of previously reported values.[10] Finally, the two Landauer functions used to describe the protonated and non-protonated states were related through the parameters κ and χ. Such that $\gamma_L^{\overline{AB}} = \kappa\gamma_L^{AB}$, $\gamma_R^{\overline{AB}} = \kappa\gamma_R^{AB}$ and $\varepsilon^{\overline{AB}} = \varepsilon^{AB} + \chi$. Therefore, only two parameters contributed to the differences in the conduction properties in the protonated and non-protonated states. The values of λ are reasonably close (within a factor of 1-2) to the values of λ determined by DFT as described in our previous report,[1] and the values of $\mathbf{E_{AB}}$ are in the range of the energy off-sets between the LUMO and the Fermi-level of the Au bottom electrode (Table S2). The other parameters have been allowed to vary for different measurements within reason (all fitting values are listed in Table S3), since the dynamic nature of the molecular junctions makes their intrinsic characteristics to change (*i.e.*, reduction state of the molecule) when V is swept within different ranges of voltage or different rates, as we describe below. This leaves us with three free fitting parameters: E_{PC}, κ and χ. As we have stated in the main text, χ represents the energy difference of the molecular level involved in the on and off state, *i.e.*, midgap state and the LUMO, and matches the computed energy difference by DFT. Γ (and thus κ) is restrained to the current that flows across the junctions

and also falls within the range of previously reported values. This leaves us only with E_{PC} as an unrestricted free fitting parameter.

Theoretical modelling of the symmetric bias cycles is complicated by the changes in oxidation state of the molecules inside the junctions, which vary with the extent of the applied bias voltage. Fig. 3a and 3b shows the experimental and theoretical (red) *I*(V) curves obtained for the bias voltage cycles (i to vii) with different voltage ranges (±0.25 to ±1.75 V) discussed in the main text. Since different applied voltage drives the junction into different oxidation states with associated different conduction states, fitting parameters that vary as a function of the oxidation state are left to vary. The parameters are given in Table S3 (identified as "Symmetric Bias Cycles"), and all lie within the range characteristic to SAM-based molecular junctions. The modelling accounts well for the overall behavior of the junction as the voltage scan range is increased, accounting for the three main characteristic responses of the junction observed in this experiment: (1) a typical reversible off-resonant tunneling behavior akin to a variable resistor for voltages cycles below ±1.0 V, (2) a molecular memory switch with a ~10^3 on/off ratio for moderate voltage cycles between ±1.0 and ±1.25 V, and (3) a negative differential resistor upon complete reduction of the HATNA states for voltages above ±1.25 V. This marked behavioral transition beyond ±1.25 V coincides with an observable variation of the fitting parameter most directly associated to the protonation process, *i.e.*, κ, which governs the off-resonance Landauer tunneling rates of the reduced molecule $\overline{AB}$. In the asymmetric negative bias experiments, κ is observed to drastically decrease when increasing the bias voltage above -1.25V (from the hundreds to the dozens). A similar effect is expected for χ, which governs the energy shift of the orbital in the reduced HATNA. However, the calculations are

insensitive to χ beyond a minimum value (~1 eV) for which the orbital is displaced beyond the range of applied voltages and therefore it does not fall inside the conduction window in our experiments. Therefore, the obtained χ values should be taken as a lower bound (*e.g.*, the orbital could be displaced to even higher energies without visibly affecting the results). Note that κ(>1) and χ are responsible for the crossing of the forward and backward *I*(V) curves observed in Fig. 3, as the overall tunneling rate and voltage at which the LUMO or midgap state enters into resonance is larger for the off state ($\Gamma_{\overline{AB}} = \kappa\Gamma_{AB}$ and $\varepsilon^{\overline{AB}} = \varepsilon^{AB} + \chi$) than the on-state. Before the midgap state reaches resonance (~ -1 V) in the on state, the current across the junction is low current, the current increases as the midgap state enters (i.e., resonance) the conduction window resulting in an increase in the current after which the current decreases when the junction switches to the off state due to reduction of HATNA. During the reverse scan, the junction stays in the off state which has a higher current than the junction in the on state for low voltages in the off-resonant tunneling regime. This sequence has been indicated by adding sequence numbers in the red arrows in Fig. 3a(vii), where steps 1 and 2 represent the on state before and after resonance, respectively, and steps 3 and 4 both represent the off state before resonance (voltages larger than -2V would have to be applied to reach the off state resonance).

Fig. 3c and 3d shows the experimental and theoretical (red) I-V curves obtained for different asymmetric negative bias cycles, where the positive voltage range is now kept constant at +1 V while the cycles are swept up to different negative voltage values ranging from 0 to -2 V. The experimental and theoretical results (see Table S2 for the corresponding fitting parameters) show the same behavior observed in the symmetric cycles shown in Fig. 3a and 3b, with the junction nicely transiting between the three characteristic regimes (*i.e.*,

variable resistor, molecular memory and NDR regimes). The observation of this multiple operative transition of the junction whether the positive bias range is kept constant (Fig. 3c) or not (Fig. S13a) confirms our interpretation that the molecular reduction occurs at negative bias, while at positive bias the molecule is oxidized back to its original molecular state.

Finally, Fig. S13 show the experimental and theoretical (red) I(V) curves obtained while keeping the maximum applied negative voltage at V = -2 V or -1.2 V for different maximum positive applied voltages. Here again the modelling provides excellent agreement with experiment with reasonable parameter values (given in Table S3 and identified as “Asymmetric Positive Bias Cycles”), which are also allowed to change because the junction samples different positive voltages in different cycles. Note the difference in the value of κ for these two measurements, small for Fig. S13a (-2 V) and large for Fig. S13c (-1.2 V), in correspondence with the variations of κ for $V >$ -1.2 V observed in the fitting of the asymmetric negative bias measurements in Fig. 3a and 3c discussed above.

Strong support for the model is given by its ability to reproduce the differences in current profiles between the first and second scans of the first cycle (i) in Fig. S13a. In this case, the junction was not pushed into a positive voltage before performing the second scan and, therefore, it remains in the reduced state achieved during the first scan and, in turn, it does not show any hysteretic or NDR behavior. Remarkably, the model captures and explains this behavior quite well. In contrast, in cycle (ii), where the junction is subjected to a small positive bias of only up to 0.25 V, the first scan is sufficient to (partially) restore the original state of the molecule, after which the junction shows a similar behavior for all

other cycles (ii to viii in Fig. S13a). Note that different positive bias ranges still have an effect on the behavior of the junction when the negative bias is kept below -1.2 V, as can be clearly observed in Fig. S13c).

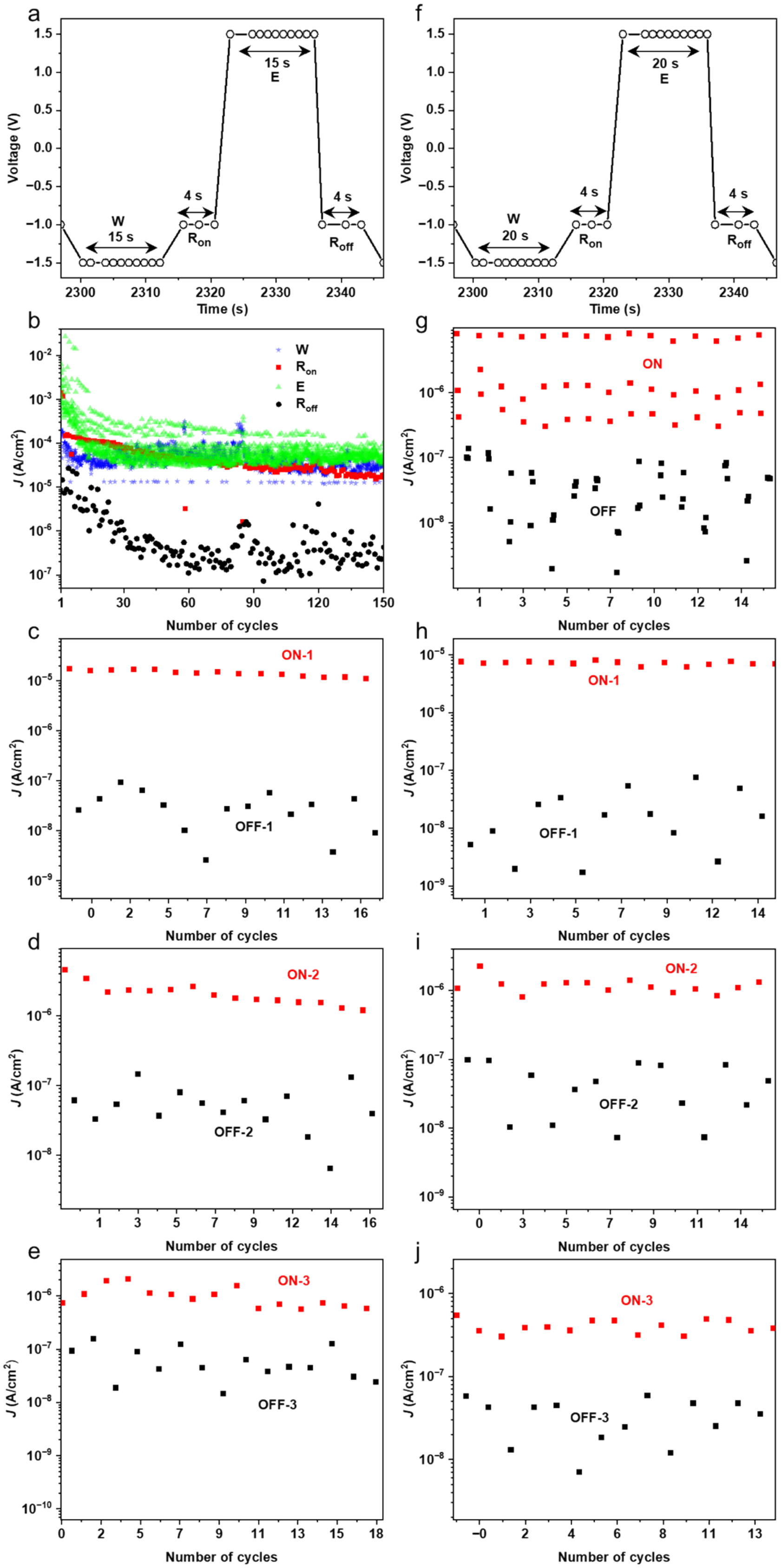
a
Voltage (V)
Time (s)
W
15 s
4 s
Ron
15 s
E
4 s
Roff
b
J (A/cm²)
Number of cycles
W
Ron
E
Roff
c
ON-1
OFF-1
d
ON-2
OFF-2
e
ON-3
OFF-3
f
20 s
g
ON
OFF
h
ON-1
OFF-1
i
ON-2
OFF-2
j
ON-3
OFF-3

Fig. S14. Switching of the junctions. (a) Write–read–erase–read voltage sequence with the applied voltage: the write voltage $V_W = +1.5$ V, the erase voltage $V_E = -1.5$ V and the read voltage $V_R = -1.0$ V, and the read time $T_R = 4$s, the write time $T_W =$ 15 s and the erase time $T_E = 15$ s; (b) the output current of the WRER measurement for the dynamic junction according to the pulse sequence in panel a; (c-e) the three different read outs current in separate for the data set as shown in main text fig. 5b; (f) Write–read–erase–read voltage sequence with the various delay time: $T_W = T_E = 20$s; (g-j) the output current of the dynamic junction according to the pulse sequence in panel f, and the three different read outs current in separate for the data set as shown in the panel g.

S7.4 Discussion.

Our model agrees very well with the experiments at both qualitative and quantitative levels providing a detailed interpretation of the reconfigurable behavior of our junction. Using what is essentially just a 2-level model, we can accurately describe this molecule as it transitions through multiple reduction states and conduction modes with only reasonable changes to the non-constant parameters. Despite the extended discussion of the model in our previous work[1], we would like to point that without the modeling we would be unable to explain the counterintuitive behavior displayed in Fig. 3 and Fig. S13. The theoretical model also helps to describe the differences between the 1st and 2nd curves of the first cycle (i) in Fig. S13a. Lastly, the model is able to show that the coupling with the electrodes changes for the protonated state as a function of the bias voltage applied. This is evident in the way κ changes with the voltage window. These results were obtained while keeping

many parameters constant throughout all of the fits and making sure that many of the others were kept in relatively close agreement.

Table S3. Parameters used to obtain the calculated *I-V* cycles shown in Fig. 3 and Fig. S13.

	**cycle**	Γ_{AB} **(meV)**	E_{AB} **(eV)**	E_{PC} **(eV)**	κ	χ **(eV)**	λ **(eV)**	γ **(s^{-1})**
**Symmetric Bias Cycles** Varying voltage (Fig. 3b)	i	0.09	0.53	-0.60	294.15	2.36	0.82	4.10
	ii	0.19	0.51	-0.76	116.35	2.86	1.15	4.74
	iii	2.34	0.61	-0.57	105.77	2.50	1.12	4.76
	iv	3.31	0.71	-1.29	170.99	0.96	1.14	4.52
	v	5.55	0.71	-1.29	171.00	0.96	1.14	4.52
	vi	13.97	0.81	-0.67	42.20	3.16	1.29	13.10
	vii	14.63	0.78	-0.64	42.21	3.27	1.30	12.85
**Asymmetric Negative Bias Cycles** Fixed positive voltage (V_{max} = +1V) (Fig. 3d)	i	0.01	0.55	-0.91	192.97	2.88	1.26	4.50
	ii	3.88	0.81	-0.55	197.38	2.55	1.20	4.50
	iii	0.13	0.66	-0.64	199.49	2.70	1.20	4.50
	iv	0.03	0.59	-0.55	25.44	2.67	1.20	4.50
	v	2.99	0.71	-1.28	171.24	1.01	1.20	4.50
	vi	4.45	0.81	-1.35	199.96	1.26	1.29	5.00
	vii	12.03	0.72	-0.85	63.30	2.61	1.13	4.64
	viii	9.49	0.69	-0.93	63.99	2.52	1.10	4.58
	ix	16.67	0.72	-1.11	37.81	2.63	1.12	3.03
**Asymmetric Positive Bias Cycles** Fixed negative voltage (V_{max} = -2V) (Fig. S13b)	i	4.80	0.68	-0.45	1.84	1.66	1.36	0.56
	ii	0.03	0.68	-0.63	6.69	0.93	1.30	1.62
	iii	0.27	0.67	-0.50	2.55	1.01	1.03	0.50
	iv	1.21	0.69	-0.49	2.18	1.01	1.03	0.47
	v	2.76	0.77	-0.55	5.56	1.00	1.01	0.43
	vi	1.71	0.84	-0.67	6.35	2.44	1.04	0.44
	vii	1.20	0.86	-1.21	1.64	2.30	0.60	5.46
	viii	2.20	0.78	-1.12	2.59	0.59	1.30	5.50
**Asymmetric Positive Bias Cycles** Fixed negative voltage (V_{max} = -1.2V) (Fig. S13d)	i	0.06	0.69	-1.29	61.03	1.11	0.72	7.55
	ii	0.01	0.47	-0.97	181.65	3.20	0.29	5.31
	iii	0.14	0.55	-0.89	181.66	2.78	0.38	5.11
	iv	3.31	0.68	-1.31	170.97	1.17	1.03	4.97
	v	3.67	0.71	-1.28	171.00	1.05	1.13	4.53
	vi	1.81	0.73	-1.29	170.93	0.97	1.16	4.17
	vii	1.16	0.74	-1.24	171.01	1.02	1.18	4.01
	viii	0.97	0.74	-1.23	171.00	1.07	1.17	4.02

References

1. Y. Wang, Q. Zhang, H. P. A. G. Astier, C. Nickle, S. Soni, F. A. Alami, A. Borrini, Z. Zhang, C. Honnigfort, B. Braunschweig, A. Leoncini, D.-C. Qi, Y. Han, E. del Barco, D. Thompson and C. A. Nijhuis, *Nat. Mater.*, 2022, **21**, 1403-1411.
2. A. Singh, D. H. Dahanayaka, A. Biswas, L. A. Bumm and R. L. Halterman, *Langmuir*, 2010, **26**, 13221-13226.
3. X. Chen, M. Roemer, L. Yuan, W. Du, D. Thompson, E. del Barco and C. A. Nijhuis, *Nat. Nanotechnol.*, 2017, **12**, 797-803.
4. L. Yuan, L. Jiang, D. Thompson and C. A. Nijhuis, *J. Am. Chem. Soc.*, 2014, **136**, 6554-6557.
5. L. Yuan, R. Breuer, L. Jiang, M. Schmittel and C. A. Nijhuis, *Nano Lett.*, 2015, **15**, 5506-5512.
6. R. C. Chiechi, E. A. Weiss, M. D. Dickey and G. M. Whitesides, *Angew. Chem. Int. Ed.*, 2008, **47**, 142-144.
7. N. Nerngchamnong, L. Yuan, D.-C. Qi, J. Li, D. Thompson and C. A. Nijhuis, *Nat. Nanotechnol.*, 2013, **8**, 113-118.
8. W. F. Reus, C. A. Nijhuis, J. R. Barber, M. M. Thuo, S. Tricard and G. M. Whitesides, *J. Phys. Chem. C* . 2012, **116**, 6714-6733.
9. A. Migliore and A. Nitzan, *J. Am. Chem. Soc.*, 2013, **135**, 9420-9432.
10. A. R. Garrigues, L. Yuan, L. Wang, E. R. Mucciolo, D. Thompon, E. del Barco and C. A. Nijhuis, *Sci. Rep.*, 2016, **6**, 26517.
